# Organ specificity in the plant circadian system is explained by different light inputs to the shoot and root clocks

**DOI:** 10.1111/nph.14024

**Published:** 2016-05-31

**Authors:** Simon Bordage, Stuart Sullivan, Janet Laird, Andrew J. Millar, Hugh G. Nimmo

**Affiliations:** ^1^ Institute of Molecular, Cell and Systems BiologyUniversity of GlasgowGlasgowG12 8QQUK; ^2^ SynthSysUniversity of EdinburghEdinburghEH9 3JDUK

**Keywords:** *Arabidopsis thaliana*, circadian rhythms, imaging, light sensitivity, organ specificity, systems biology

## Abstract

- Circadian clocks allow the temporal compartmentalization of biological processes. In Arabidopsis, circadian rhythms display organ specificity but the underlying molecular causes have not been identified. We investigated the mechanisms responsible for the similarities and differences between the clocks of mature shoots and roots in constant conditions and in light : dark cycles.
- We developed an imaging system to monitor clock gene expression in shoots and light‐ or dark‐grown roots, modified a recent mathematical model of the Arabidopsis clock and used this to simulate our new data.
- We showed that the shoot and root circadian clocks have different rhythmic properties (period and amplitude) and respond differently to light quality. The root clock was entrained by direct exposure to low‐intensity light, even in antiphase to the illumination of shoots. Differences between the clocks were more pronounced in conditions where light was present than in constant darkness, and persisted in the presence of sucrose. We simulated the data successfully by modifying those parameters of a clock model that are related to light inputs.
- We conclude that differences and similarities between the shoot and root clocks can largely be explained by organ‐specific light inputs. This provides mechanistic insight into the developing field of organ‐specific clocks.

## Introduction

Circadian clocks are biological oscillators that have evolved in most organisms in response to the daily rotation of the earth; they drive rhythms at the molecular and cellular levels and thus temporally regulate many aspects of physiology and behaviour to anticipate changes in the environment. Circadian rhythms are generated endogenously, persist in constant conditions, have a period close to 24 h and can be entrained by zeitgebers such as light : dark (LD) or warm : cold cycles. In Arabidopsis, expression of about one‐third of the genome is under circadian regulation (Michael & McClung, 2003; Covington *et al*., 2008) and at the physiological level the circadian clock can control many plant processes, including photosynthesis, leaf movement, hormone responses, stem extension and stomatal opening (McClung, 2006; Harmer, 2009; Pruneda‐Paz & Kay, 2010). Appropriate circadian timing can confer a competitive advantage (Green *et al*., 2002; Dodd *et al*., 2005).

Conceptually, the circadian clock can be considered to comprise a core oscillator with input pathways that allow entrainment to the environment and output pathways that determine the timing of physiological rhythms. Based on experimental studies, mainly of seedlings, and on modelling, the Arabidopsis core oscillator includes multiple interlocking feedback loops of gene expression, modulated by posttranslational control at several levels (Harmer, 2009; Nagel & Kay, 2012; Hsu & Harmer, 2014; Millar, 2016). Key players include the morning‐expressed MYB transcription factors CIRCADIAN CLOCK ASSOCIATED 1 (CCA1) and LATE ELONGATED HYPOCOTYL (LHY), the day‐phased transcriptional regulators PSEUDO‐RESPONSE REGULATOR 9 (PRR9) and PRR7 and the evening‐phased components GIGANTEA (GI) and PRR1 (also known as TIMING OF CAB EXPRESSION 1 (TOC1)). In early formalizations of the clock, these and other components were arranged in interconnected ‘morning’, ‘central’ and ‘evening’ loops (Locke *et al*., 2006; Zeilinger *et al*., 2006). However, additional experimental and theoretical work led to development of a more complex network including a ‘repressilator’ circuit (Pokhilko *et al*., 2012).

Early work suggested that plant cells contain independent and autonomous copies of the clock (Thain *et al*., 2000), although some tissue specificity of period was noted (Thain *et al*., 2002). Exposure of seedling roots to LD cycles entrained them independently of shoots (Thain *et al*., 2000). However, roots are not usually exposed to direct light; we therefore compared the root and shoot clocks in hydroponically grown plants with the roots in darkness (James *et al*., 2008). Using quantitative real‐time (RT‐q)PCR measurement of gene expression, we showed that the free‐running period (FRP) of the root clock in constant light (LL) was longer than that in shoots, although the organs were synchronized in LD, suggesting that roots can be entrained by a signal from shoots. We found no evidence for rhythmic expression of the clock evening‐phased genes in wild‐type roots under LL, though oscillations were detected in *prr7,9* mutant roots, and we concluded that the root clock was a simplified slave version of the shoot clock. Recent work has focussed on the potential for hierarchical coupling of tissue‐specific clocks. The proposed vascular‐specific expression of *PRR3* (Para *et al*., 2007) might be part of a distinct vascular clock that can influence the mesophyll clock (Endo *et al*., 2014). Takahashi *et al*. (2015) showed that the clocks in shoot apex cells are coupled by intercellular communication and can send a signal to roots that maintains circadian synchrony within roots, such that root rhythms damp rapidly after excision of the apex; they also confirmed the period difference between shoots and roots.

The microenvironments of roots and shoots are very different. Nevertheless, dark‐grown roots can perceive a fraction of the light illuminating shoots, via light piping along plant tissue (Mandoli & Briggs, 1984; Sun *et al*., 2003, 2005) or the limited penetration of light into soil (Tester & Morris, 1987). This raises the question of whether the observed differences between the shoot and root clocks are attributable mainly to different environmental conditions or to intrinsic differences between the two tissues. To address this question and to define the properties of the root clock more fully, we developed the ability to monitor luciferase activity as a reporter in shoots and roots separately of plants whose roots were covered or exposed to the prevailing LD cycle.

In this work, we confirmed and further defined the organ specificity of the plant circadian system: the root and shoot clocks behaved differently when light was present, in LD or LL, but were similar in constant darkness (DD). Using a combination of experimental and theoretical approaches, we showed that the main differences observed between the shoot and root clocks persist in the presence of sucrose and can be explained by different light inputs. By modifying light‐related processes in a recent mathematical model of the shoot clock, we simulated both the main differences between the shoot and root clocks and their similarities such as entrainment by light. Some predictions of this new root model were verified experimentally, and by using excised root tissues we showed that the root clock can be entrained by direct perception of very low intensity light.

## Materials and Methods

### Plant material and growth

Surface sterilized seeds of *Arabidopsis thaliana* (L.) Heynh. were stratified for 2–4 d at 4°C before sowing. Seeds for imaging were sown on 1.2% agar in 0.5 strength Murashige and Skoog (MS) medium adjusted to pH 5.7 in 120‐mm square vertical plates. Ten to twelve days after germination, seedlings (two clusters of three plants per plate) were transferred to fresh plates in which the top 3 cm had been replaced with 1.8% agar and 2% charcoal in 0.5 strength MS medium, readjusted to pH 5.7 after addition of charcoal. For dark‐grown roots, the shoot and root compartments were separated by a black bar and the root compartment was covered with black tape (Supporting Information Fig. S1); however, dark‐grown roots may perceive some light through light piping or by leakage from the shoot compartment. After a further 10–12 d, plants were sprayed with luciferin and the plates were sealed with new lids containing a black barrier to separate the shoot and root compartments (Fig. S1). Hydroponic culture was essentially as described previously (James *et al*., 2008), except that plants were harvested between 4 and 5 wk after sowing. Tissue was collected under a green safety light, immediately frozen in liquid nitrogen and stored at −80°C until use. For light‐grown roots, black boxes were replaced by transparent boxes and additional lights were placed at the bottom of the growth cabinet so that roots were exposed to the same light intensity as shoots. Plants were grown under white light (100 μmol m^−2^ s^−1^) in 12 h : 12 h, light : dark cycles at 20°C for 3–4 wk from sowing to harvesting or imaging.

The [clock gene promoter]:LUC+ (luciferase) fusions were in the Ws (Wassilewskija) background of Arabidopsis unless stated otherwise. The CCA1:LUC+, TOC1:LUC+, PRR9:LUC+, GI:LUC+ and COLD, CIRCADIAN RHYTHM AND RNA BINDING PROTEIN 2 (CCR2):LUC+ expressing lines have been described previously (Doyle *et al*., 2002; McWatters *et al*., 2007; Kim *et al*., 2008; Edwards *et al*., 2010). The *cca1‐11,lhy‐21* double mutant expressing CCR2:LUC+ was obtained from the Nottingham Arabidopsis Stock Centre (N9809). PRR7:LUC+ expressing lines and the *toc1‐4* mutant expressing CCR2:LUC+ are in the Columbia (Col‐0) background (Salome & McClung, 2005; Para *et al*., 2007).

### Luciferase imaging

Three‐ to four‐week‐old plants were sprayed with 60 mM D‐luciferin in 0.01% triton (300 μl per plate). Dark‐grown roots were sprayed under low‐intensity green light and then kept in the dark by an automated system in which a tight‐fitting black cover supported on runners could be moved across the root compartments as required. Plates were kept at 20°C and illuminated by equal amounts of blue and red light provided by LEDs at a total intensity of 15 μmol m^−2^ s^−1^ unless stated otherwise. Bioluminescence was detected using a Photek 225/18 Intensified CCD camera with a 16‐mm lens (Photek Ltd, St Leonards on Sea, UK). The camera, LEDs and covering system were controlled using Photek ifs32 software. Images (15 min) were recorded every 1.5–3 h in photon counting mode, without any filters. Root and shoot regions were defined and luminescence data extracted using Photek ifs32 software. For each cluster of plants, the luminescence at each time‐point was mean normalized to the average luminescence over the corresponding time‐course in order to control for the different amounts of tissue exposed to the camera in these clusters.

### RNA extraction and quantification by RT‐qPCR

RNA extraction, cDNA synthesis and qPCR were performed as described previously (James *et al*., 2008), with modifications. Total RNA was extracted with the RNeasy Plant Mini kit (Qiagen) and DNase treated (DNA‐free; Ambion). Absence of genomic DNA contamination was confirmed by PCR with *ACTIN2* gene primers. Complementary DNA (cDNA) was synthesized from 1 μg of total RNA using oligo dT and SuperScript II reverse transcriptase (Invitrogen). qPCR reactions were performed with Brilliant III SYBR Green QPCR Master Mix (Agilent) on a Mx3000P (Agilent Technologies Ltd, Stockport, UK) or a StepOnePlus (Fisher Scientific‐UK Ltd, Loughborough, UK) real‐time PCR system. *IRON SULFUR CLUSTER ASSEMBLY PROTEIN 1* (*ISU1*, At4G22220) was used as the reference gene as its expression has been shown not to cycle over a range of conditions (Michael *et al*., 2008). All primer sequences are given in Table S1.

### Data analysis

Mean normalized time‐course data from imaging and qPCR experiments were analysed using Biological Rhythm Analysis Software System (brass) (Edwards *et al*., 2010; www.amillar.org) without prior detrending, with data from the first 24 h in constant conditions discarded. Period, amplitude and relative amplitude error (RAE) were analysed using the fft‐nlls algorithm within brass. Amplitudes from normalized data are referred to as ‘relative amplitudes’ hereafter. The RAE is the ratio of the amplitude error to the most probable amplitude. It assesses rhythm robustness: values close to 0 and 1 indicate robust and weak (if any) rhythms, respectively. One‐way analysis of variance (ANOVA) was carried out with sigmaplot 11.0 (Systat Software Inc., London, UK). Significance was determined by using the Tukey test for multiple pairwise comparisons.

### Modelling

We modelled the behaviour of roots using a recent mathematical model of the Arabidopsis shoot clock (Pokhilko *et al*., 2012), termed the P2011 model, accessed from the Plant Systems Biology Modelling database (http://www.plasmo.ed.ac.uk). We used copasi (v.4.8.35, http://www.copasi.org) to explore manually parameters related to light inputs and to simulate the values of appropriate components as time‐courses. Parameters were initially varied stepwise over a range from 10‐fold below to 10‐fold above the default values (i.e. those in the P2011 model), either alone or in combination, then in more detail from two‐fold below to two‐fold above the default values. Data were inspected to select the best match to the following constraints (ranked in order of decreasing importance): longer periods in light‐ and dark‐grown roots compared to shoots under LL, lower amplitudes in dark‐grown roots compared with shoots under LD, and higher trough levels for evening genes in dark‐grown roots compared with shoots. Full details are given in Methods S1.

## Results

### Light can directly affect clock gene expression in roots

**Figure 1:**
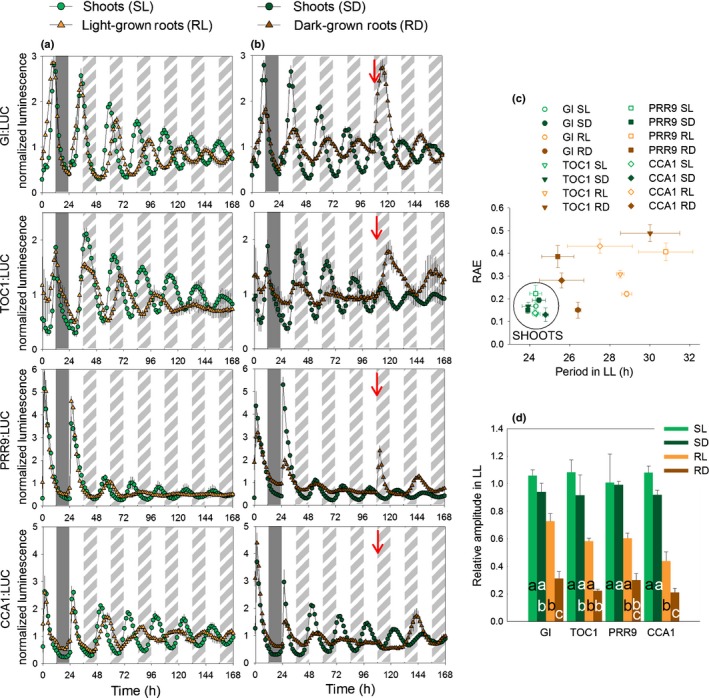
The expression of clock genes in roots is affected by direct exposure to light. *Arabidopsis thaliana* plants carrying a [clock gene promoter]:LUC+ fusion were entrained for 3–4 wk in light : dark (LD 12 h : 12 h) before release in light : light (LL). Plants with (a) light‐grown and (b) dark‐grown roots were monitored over the last day in LD (time 0 = dawn) and in LL. From 108 h (red arrows in b), dark‐grown roots were exposed to the same light conditions as shoots and light‐grown roots. For each experiment, one to three clusters of two to six plants (organs) were imaged separately and luminescence data were normalized to the mean values between times 0 and 108 h. Error bars are means ± SE for three independent experiments. The backgrounds show days or subjective days (white bars), night (dark grey bars) and subjective night (hatched bars). (c) Circadian periods and (d) relative amplitudes were estimated between times 48 and 108 h for individual time‐courses (S, shoots; R, roots; L, light‐grown roots; D, dark‐grown roots). Relative amplitudes were normalized to the mean relative amplitude of the shoots (SL and SD) for each reporter. Error bars are SEM. Values in (d) are statistically different (*P *<* *0.05, by one‐way ANOVA) if they do not have any letter in common. RAE, relative amplitude error.

To obtain high‐resolution data on the circadian clock in mature tissues, we developed a system (see the Materials and Methods section; Fig. S1) to monitor luciferase activity in the shoots and roots separately of plants grown on vertical agar plates. The roots were exposed to the prevailing light : dark cycle (light‐grown roots) or covered to mimic physiological conditions more closely (dark‐grown roots). Plants expressing one of four different promoter:LUC+ constructs (for the morning‐expressed genes *CCA1* and *PRR9* and the evening‐expressed genes *TOC1* and *GI*) were monitored over one LD cycle followed by 6 d in LL. Fig. 1(a) shows data for the shoots and roots of plants with light‐grown roots, while Fig. 1(b) shows the corresponding data for plants with dark‐grown roots. Shoots behaved consistently irrespective of whether the roots were illuminated or not (Fig. 1c,d). Root rhythms were less robust and the periods tended to be more variable between experiments (Fig. 1c; Table S2). For example, in dark‐grown roots, the TOC1:LUC+ and CCA1:LUC+ plants were rhythmic only in some data sets. The high RAE values indicate weak rhythmicity for these markers (Fig. 1c) and the time‐series data show that the rhythms damped out by 72 h in LL (Fig. 1b). However, the FRPs of morning and evening clock genes were consistently longer in both light‐grown roots and dark‐grown‐roots than in shoots. Long FRPs in both dark‐ and light‐grown roots were also observed for the promoter activity of an output gene (*CCR2*; Fig. S2); therefore, the difference in FRP between shoots and roots was not eliminated simply by illuminating the roots.

Nevertheless, light affected clock gene expression in roots. The relative amplitudes of clock gene oscillations were higher in shoots and light‐grown roots than in dark‐grown roots under LL (Fig. 1a,b (before 108 h), d). Interpretation of amplitudes in LL is complicated by the fact that loss of synchrony within an organ can lead to damping (Fukuda *et al*., 2012). However, lower amplitudes in dark‐grown roots were also observed in LD, using both imaging and qPCR (Figs 1a,b, S3). Exposing dark‐grown roots to light at 108 h (from the red arrows in Fig. 1a,b) rapidly induced activity of the *PRR9* promoter followed by the *GI*,* TOC1* and *CCA1* promoters. The total luminescence (i.e. before normalization) was comparable in light‐ and dark‐grown roots after 108 h, when dark‐grown roots were exposed to the same light conditions as light‐grown roots (Fig. S4). Exposing roots to light did not significantly affect the relative amplitude or FRP of clock genes in shoots (Fig. 1c,d).

**Figure 2:**
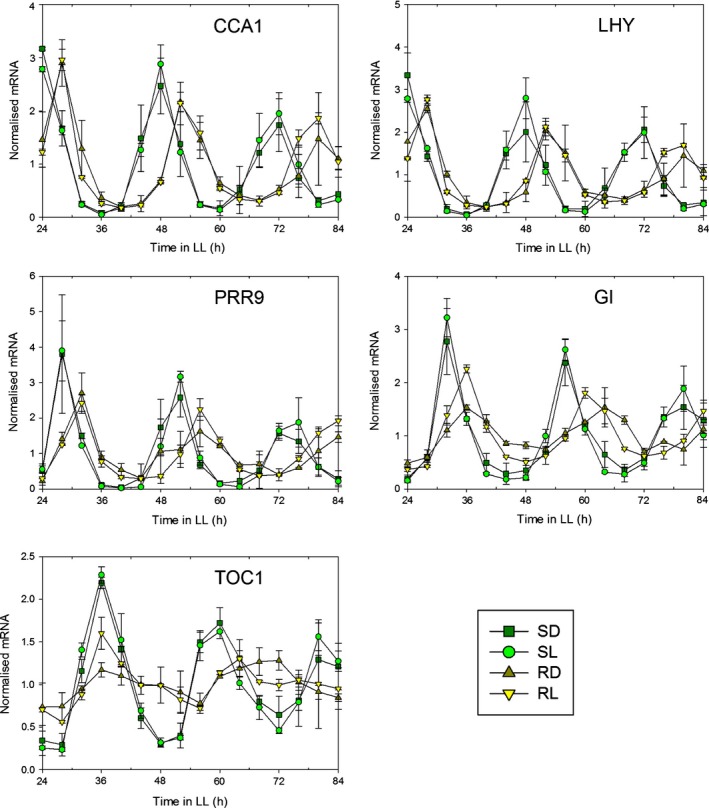
Light affects the relative amplitude but not the free‐running period (FRP) of clock gene expression in roots under light : light (LL). Wild‐type *Arabidopsis thaliana* plants were grown hydroponically under light : dark (LD 12 h : 12 h) with roots kept in the dark. After 3 wk of entrainment, half of the plants were transferred into transparent boxes so shoots and light‐grown roots were equally illuminated. Plants were grown for another week in LD before transfer to constant light and harvesting. Gene expression was analysed by quantitative real time (RT‐q)PCR and expressed relative to IRON SULFUR CLUSTER ASSEMBLY PROTEIN 1. R, roots; S, shoots; L, transparent boxes; D, black boxes. Data are mean ± standard deviation for two biological replicates. All genes shown were scored as rhythmic by Biological Rhythm Analysis Software System (brass); mean circadian periods are shown in Supporting Information Table S3.

The observed rhythmicity in roots of all of the clock gene promoters tested in at least some data sets contrasts with the RT‐qPCR data of James *et al*. (2008), who found that only the morning loop genes were scored as rhythmic in roots of plants under LL. The discrepancy was not caused by the difference in ecotype (Ws in Fig. 1, and Col‐0 in our earlier RT‐qPCR experiments) because the bioluminescence of TOC1:LUC in dark‐grown roots was also scored as rhythmic in Col‐0 (Fig. S5). We therefore used an improved protocol for RT‐qPCR analysis to measure transcript abundances in plants grown hydroponically in either black or transparent boxes, giving dark‐ or light‐grown roots, respectively. Fig. 2 shows that both morning‐ and evening‐expressed genes were rhythmic in roots, though the rhythms were less robust than the shoot rhythms. The periods observed for light‐ and dark‐grown roots were similar and were consistently greater than the corresponding values for shoots. Furthermore, the relative amplitudes of rhythms in light‐grown roots were greater than those in dark‐grown roots (Table S3).

Thus, these data confirm and extend the organ specificity of the plant circadian system (James *et al*., 2008): the root clock has a longer FRP under LL than the shoot clock irrespective of whether roots are illuminated. In addition, the relative amplitude of clock gene expression is lower in dark‐grown roots compared with shoots or light‐grown roots, and direct exposure to light can increase relative amplitude in roots. Hence, oscillations in both promoter strength and transcript abundance can be detected more easily for the clock evening‐expressed genes in roots either if the roots are illuminated, which increases relative amplitude, or if luciferase imaging is used, which provides increased time resolution and avoids inter‐sample variation among time‐points.

### Evening‐expressed genes are functionally important in the root clock

**Figure 3:**
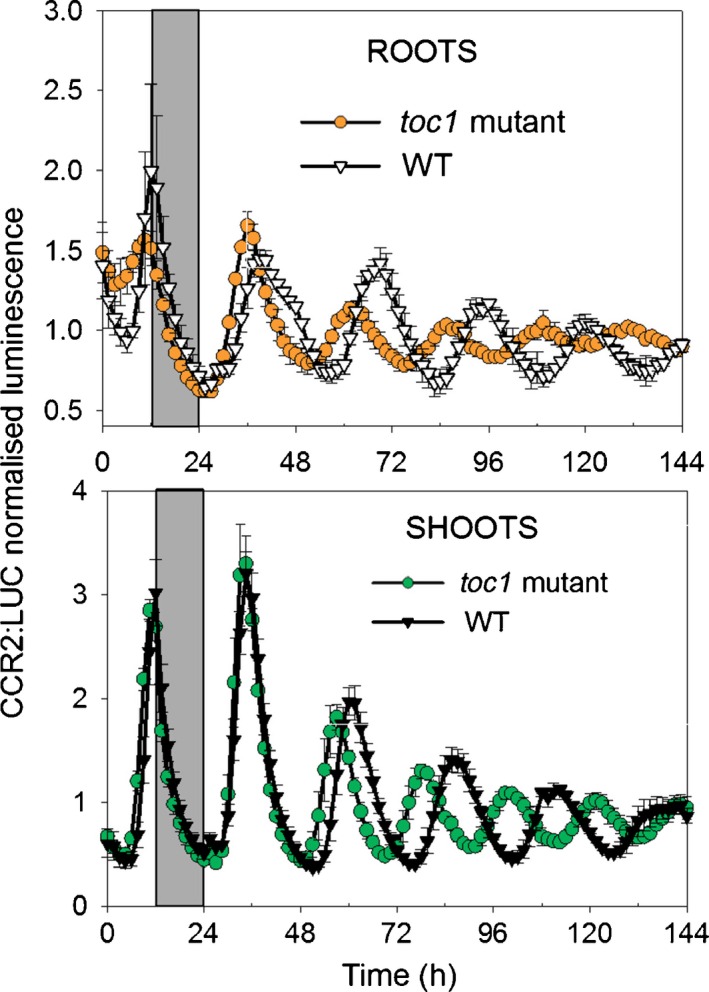
*TIMING OF CAB EXPRESSION 1* (*TOC1*) affects the dynamics in both shoot and root clocks in light : light (LL). *Arabidopsis thaliana* plants with dark‐grown roots were grown for 4 wk in light : dark (LD) before transfer to LL. *toc1‐4* mutant and wild‐type (WT) plants expressing COLD, CIRCADIAN RHYTHM AND RNA BINDING PROTEIN 2:LUCIFERASE (CCR2:LUC+) were imaged over the last day in LD (time 0 = dawn) and in LL for roots and shoots. The black bars in the backgrounds represent the last night cycle. Error bars are ± SEM for three independent experiments.

Using RT‐qPCR, no effect of the *toc1‐10* mutation on circadian period in roots was detected (James *et al*., 2008). We exploited the advantages of the luciferase assay to reinvestigate the functional importance of TOC1 in the root clock. Plants expressing the CCR2:LUC reporter were imaged for one LD cycle followed by 120 h in LL. Fig. 3 shows that the *toc1‐4* mutation shortened the period of rhythms in both roots and shoots by 3–4 h. The rhythmicity of *GI* in roots is evident from Figs 1 and 2. Mutations in another evening‐expressed clock gene, *EARLY FLOWERING 3* (*ELF3*), have already been shown to affect circadian rhythmicity in roots (Takase *et al*., 2011). Thus, it is reasonable to conclude that these evening‐expressed genes contribute to the function of the root clock, even though TOC1:LUC+ roots are rhythmic only in some data sets. Our imaging also provided data consistent with the known effects of other mutations; for example, we detected a shortening of period in both shoots and roots of the *cca1‐11,lhy‐21* mutant compared with the wild‐type (Fig. S6a).

### The root clock can be entrained by direct perception of LD cycles

**Figure 4:**
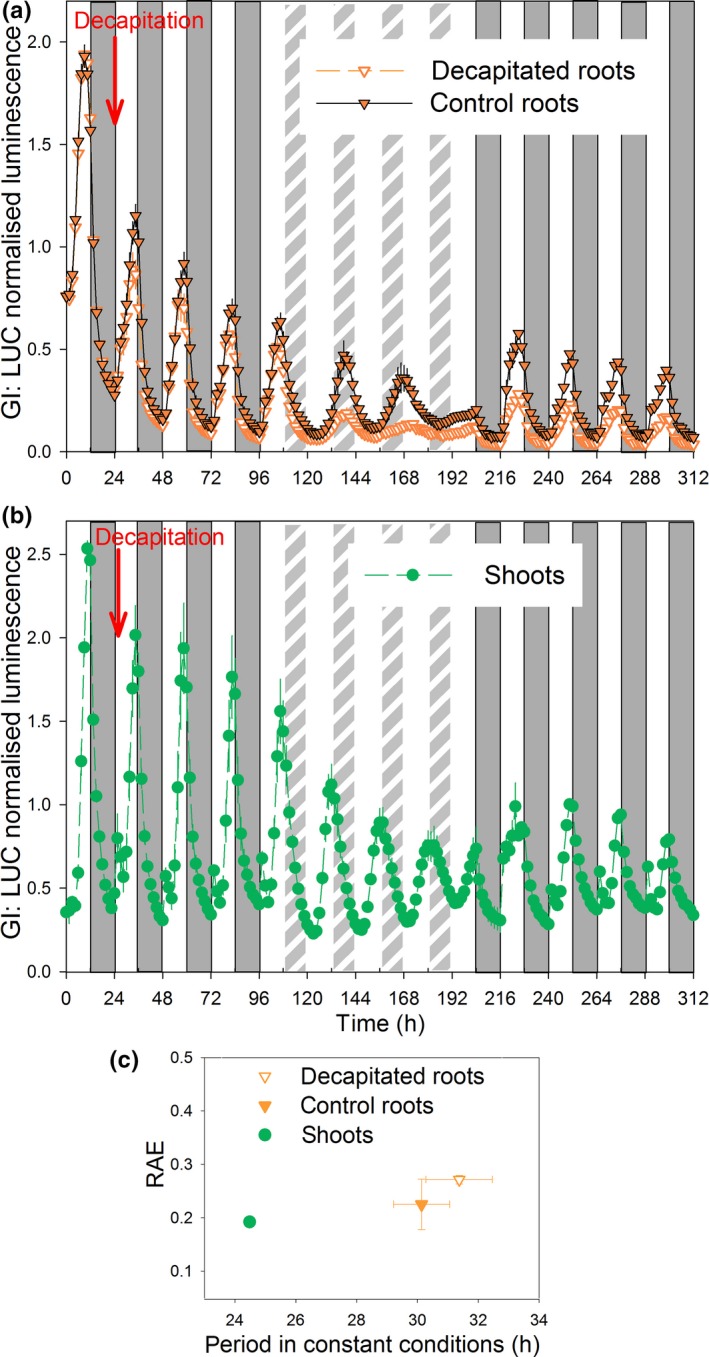
Roots can be entrained by direct perception of light : dark (LD) cycles. *Arabidopsis thaliana* plants with the *GIGANTEA:LUCIFERASE* (*GI:LUC*+) reporter were entrained for 3–4 wk in LD (with light‐grown roots) before imaging. Plants were imaged for 24 h in LD before one set of plants were decapitated before dawn (at time 24 h, indicated by the red arrows). Light‐grown (a) roots and (b) shoots were imaged over a further 72 h in LD, 96 h in light : light (LL) and 120 h in LD. Grey bars represent dark cycles; white and light grey hatched bars represent light and subjective light, respectively. Data were normalized to the mean luminescence of the first LD cycle (before decapitation). Error bars are ± SEM for four clusters of two to three plants from three independent experiments, except for the last 3.5 LD cycles (following the LL period) where only one cluster was imaged. (c) Periods were estimated from the LL data shown in (a) and (b). RAE, relative amplitude error.

Previous work suggested that the clock of dark‐grown roots was a slave oscillator entrained indirectly through a rhythmic signal, possibly sucrose, from shoots in plants grown under LD cycles (James *et al*., 2008). However, the present study shows that the root clock is influenced by direct exposure to light. To test whether light alone can entrain roots, we studied the effects of decapitation (i.e. removal of the shoots) on plants expressing the GI:LUC+ reporter gene. Plants with light‐grown roots were first entrained in LD cycles for 3 wk, and then imaged for 4 d in LD, 4 d in LL and another 5 d in LD. Half of the plants were decapitated at the end of the first night of imaging (time 24 h in Fig. 4). Both control and decapitated roots were entrained to the initial LD cycles, ran free in LL and then became re‐entrained in LD; levels of luminescence were lower in decapitated roots compared with controls (Fig. 4a). The periods of *GI* expression in roots were *c*. 24 h under LD and 30–32 h under LL (Fig. 4a,c); decapitation had little effect on the root period. The control shoots were entrained under LD cycles and ran free in LL as expected (Fig. 4b,c). Thus, *GI* expression can be entrained by direct exposure of roots to light.

The sensitivity of roots to light is illustrated in Fig. S7. Plants with dark‐grown roots expressing GI:LUC+ were imaged for 24 h in LD, 48 h in DD, over four LD cycles at 0.15 μmol m^−2^ s^−1^ and finally in DD. Some plants were decapitated 24 h before the start of imaging. The data show that the roots, both decapitated and from intact plants, were strongly entrained by the very low intensity light and then free ran in the final DD period.

**Figure 5:**
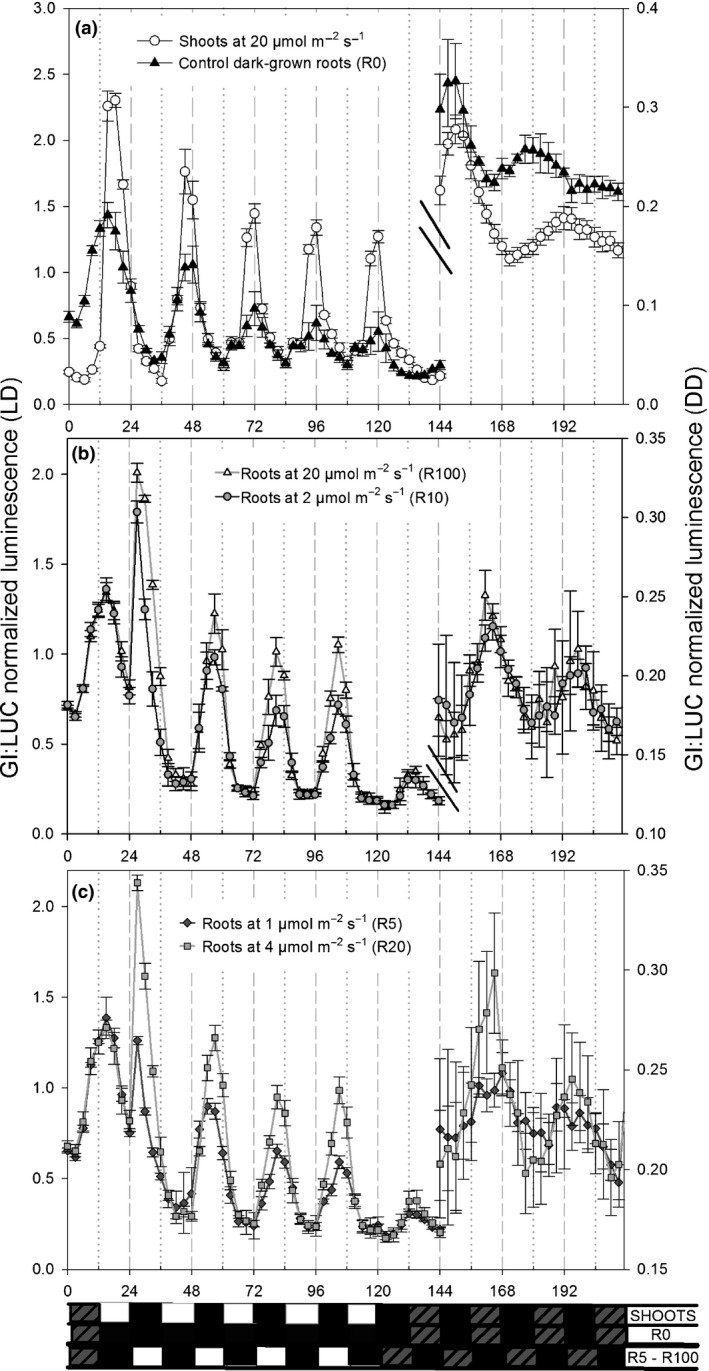
Roots are entrained by direct perception of light in preference to putative rhythmic signals from shoots. *Arabidopsis thaliana* plants with dark‐grown roots expressing GIGANTEA:LUCIFERASE (GI:LUC+) were entrained for 3 wk in light : dark (LD), sprayed with luciferin at dusk, then kept in darkness for 24 h; imaging commenced after 12 h of this dark period. The shoots were then exposed to LD cycles (20 μmol m^−2^ s^−1^ of red + blue light) in antiphase to the cycles before imaging. The roots were kept in the dark for a further 12 h and then exposed to LD cycles (1–20 μmol m^−2^ s^−1^ of red + blue light); control roots were kept in the dark. Data are mean ± standard deviation for at least three clusters of plants in two to three independent experiments. (a) Shoots and control roots; (b) roots at 2 or 20 μmol m^−2^ s^−1^; (c) roots at 1 or 4 μmol m^−2^ s^−1^. DD, dark : dark.

To investigate the relative strengths of direct perception of light and shoot‐to‐root signals, we exposed shoots and roots to conflicting light : dark cycles at different intensities. After growth in LD, shoots were kept in darkness for 24 h, then exposed to LD cycles (20 μmol m^−2^ s^−1^ of red + blue light) in antiphase to the cycles before imaging. The roots were kept in darkness for 36 h, then exposed to LD cycles (1–20 μmol m^−2^ s^−1^ of red + blue light) with controls kept in the dark. Shoots and control dark‐grown roots behaved similarly (Fig. 5a); after two transient cycles (time 12–60 h), they were synchronously entrained to the new LD cycle (time 60–132 h), with peak expression of *GI* at dusk and a period of *c*. 24 h in both organs. By contrast, the roots exposed to light rapidly became entrained to their new LD cycle and were thus in antiphase to their shoots; expression of *GI* oscillated with a peak *c*. 3 h before dusk and period *c*. 24 h (Fig. 5b,c). These results confirm that the circadian clock in roots is directly sensitive to light, and show that even at 1 μmol m^−2^ s^−1^ roots respond directly to an LD cycle in preference to any putative rhythmic signal translocated from shoots to roots.

**Figure 6:**
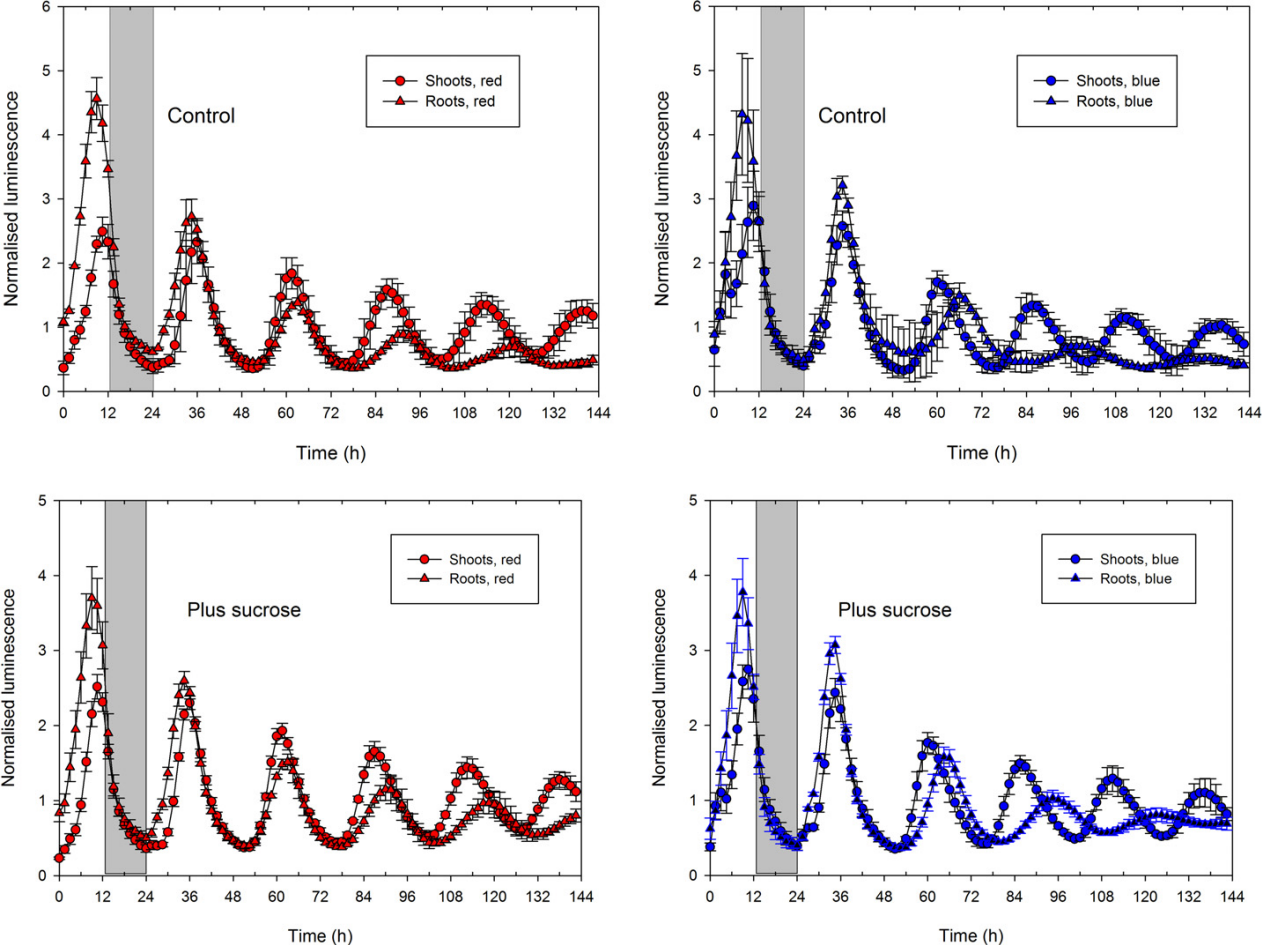
Effect of light quality on period. *Arabidopsis thaliana* plants with light‐grown roots expressing GIGANTEA:LUCIFERASE (GI:LUC) were entrained for 3 wk in light : dark (LD). Half of the plants were transferred to standard imaging plates and half to plates containing 1% sucrose. Plants were imaged for 24 h in LD followed by 120 h in light : light (LL), using either red or blue light at 15 μmol m^−2^ s^−1^. Data are mean ± standard deviation for *n *=* *4 clusters of plants from two biological replicates. Periods are given in Supporting Information Table S4.

To compare further the effects of light and sucrose on the root clock, we monitored rhythms in LL in the presence or absence of 1% sucrose, using equal intensities of red or blue light. The results (Fig. 6; Table S4) show that the difference in period between shoots and roots was maintained in the presence of sucrose; furthermore, the root period but not the shoot period was strikingly longer in blue light than in red light, whether or not sucrose was present.

### The shoot and root clocks have similar dynamics in constant darkness

Having established differences between the shoot and root clocks in plants exposed to light, we used imaging to investigate clock dynamics in DD. Rhythms damped rapidly, as expected from the work of Dalchau *et al*. (2011), who noted that rhythmicity in DD was difficult to detect without sucrose in the medium. Overall, some 65% of plants expressing GI:LUC+ were scored as rhythmic; the periods were variable, between *c*. 30 and *c*. 36 h, but there was no significant difference between roots and shoots (Fig. S8).

We investigated the properties of rhythms in DD in two further ways. RT‐qPCR time‐courses for *CCA1*,* LHY*,* PRR9*,* GI* and *TOC1* were scored as rhythmic (Fig. S9; Table S5), although amplitudes declined markedly between the first and second cycles in DD. The periods estimated by RT‐qPCR were appreciably shorter than those from imaging, probably because of the substantial differences in experimental conditions required for the two approaches. However, for each method there was no difference in period between shoots and roots. We also monitored rhythmicity in plants expressing GI:LUC+ grown on plates containing 1% sucrose. Roots and (to a lesser extent) shoots displayed robust rhythms in these conditions (Fig. S10); there was no significant difference in period but the phase of the shoot rhythms was slightly delayed relative to the root rhythms. It is clearly difficult to estimate periods precisely in DD but overall the data indicate that there is little difference between the shoot and root clocks in these conditions.

### Differences in light inputs can account for differences between the shoot and root clocks

Our experimental data show that the shoot and root core clocks were more similar in DD than LL: the main differences between shoots and roots, in period and amplitude, were observed when light was present, irrespective of whether the medium contained sucrose. In addition, roots were directly sensitive to low light intensities, and the shoot and root clocks responded differently to light quality. We therefore hypothesized that differences between the shoot and root circadian systems are attributable to organ‐specific light input pathways.

**Figure 7:**
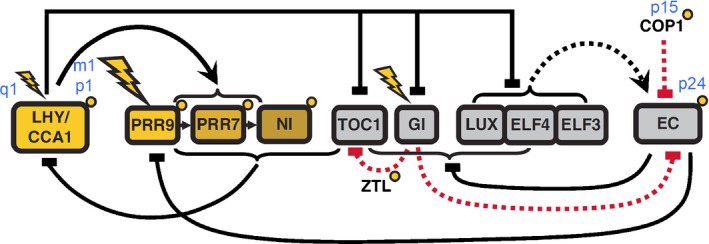
Diagrammatic representation for the P2011 model of the Arabidopsis circadian clock. Only the main elements – genes (boxed), transcriptional regulation (solid lines) and the location of light inputs – are shown. Acute light responses in gene transcription are shown by flashes. Posttranslational regulation by light is shown by small yellow circles. Elements of the morning and evening loops are in yellow and grey boxes, respectively. The positions and names of parameters that were modified for the root model are also shown in blue. Proteins are shown only for evening complex (EC), ZEITLUPE (ZTL) and CONSTITUTIVE PHOTOMORPHOGENIC 1 (COP1) for simplicity. The EC protein complex formation is denoted by a dashed black line. Posttranslational regulation of TOC1 and EC by GI, ZTL and COP1 is shown by red dashed lines. The diagram was adapted from Pokhilko *et al*. (2012).

To test this hypothesis, we used the P2011 model (Fig. 7), whose default values gave an accurate description of our shoot data, and we modified processes controlled by light that might be altered in roots. Our objective was to simulate the observed differences in clock behaviour between shoots and roots in LL and LD but also their similar behaviour in DD. Light‐related processes are either transiently or permanently affected by light (represented in Fig. 7 by flashes and small yellow circles, respectively). Both are controlled by the parameter *L* which by default is 1 when light is present and 0 during dark cycles. Simulating lower light intensity by reducing the value of *L* recapitulates the longer period in LL of dark‐grown roots compared with shoots (Fig. S11), illustrating that the P2011 model is consistent with Aschoff's rule (Aschoff, 1960). It also captures the lower amplitude of dark‐grown roots than shoots under LD. However, this does not explain why light‐grown roots exhibit a longer FRP than shoots exposed to the same light conditions, but also have higher amplitude rhythms than dark‐grown roots (Fig. 1). These features could be attributable to differential effects of light on clock processes in shoots and roots. In this section, we varied the values of appropriate parameters of the P2011 model by 10‐fold above and below the default values (see the Materials and Methods section) and report the values that provided the best fit to our experimental data.

In the P2011 model, processes that mediate acute effects of light in LD have no effect in LL. We therefore started by simulating the differences in clock behaviour between shoots and roots in LD. We noted that the expression of *CCA1* was not acutely induced by light in roots (Fig. 1). This behaviour was best simulated by halving the value of *q*_1_ which represents the acute induction of *CCA1*/*LHY* mRNA by light in the P2011 model, where *LHY* and *CCA1* are modelled by a single component. In addition this parameter change also gave a more accurate (i.e. earlier) phase for *CCA1*/*LHY* under LD (Fig. S11).

We then simulated the long periods observed in roots in LL by modifying processes that are permanently affected by light. Some of these could also affect the levels of CCA1/LHY and evening complex (EC) proteins through the parameters *p*_1_, *p*_15_ and *p*_24_. These parameters are rate constants for light‐dependent processes (*p*_1_: translation of CCA1/LHY; *p*_15_: translocation of CONSTITUTIVE PHOTOMORPHOGENIC 1 (COP1) between nucleus and cytoplasm; *p*_24_: modification of EC). We reasoned that reducing the peak levels of the EC and CCA1/LHY proteins would also increase the trough levels of evening gene mRNAs as observed experimentally (Fig. 2). Both increased trough levels and lengthened FRP were satisfactorily achieved by halving the values of *p*_*1*_ and *p*_*15*_ and by increasing the value of *p*_*24*_ by 50% for roots compared with the default values representing shoots.

The new parameter values for light‐ and dark‐grown roots compared with shoots are summarized in Table S6. The changes in *q*_*1*_, *p*_*1*_, *p*_*15*_ and *p*_*24*_ were used to simulate both light‐ and dark‐grown roots. The distinction between these was achieved by reducing the value of *L* from 1 to 0.5 for dark‐grown roots. To increase the FRP in light‐grown roots further so that it became similar to the FRP of dark‐grown roots, the value of *m*_*1*_ was decreased by 25% for light‐grown roots only. None of these changes influenced clock dynamics under DD (when *L* is 0), consistent with the similarities observed between shoot and root clocks in constant darkness.

**Figure 8:**
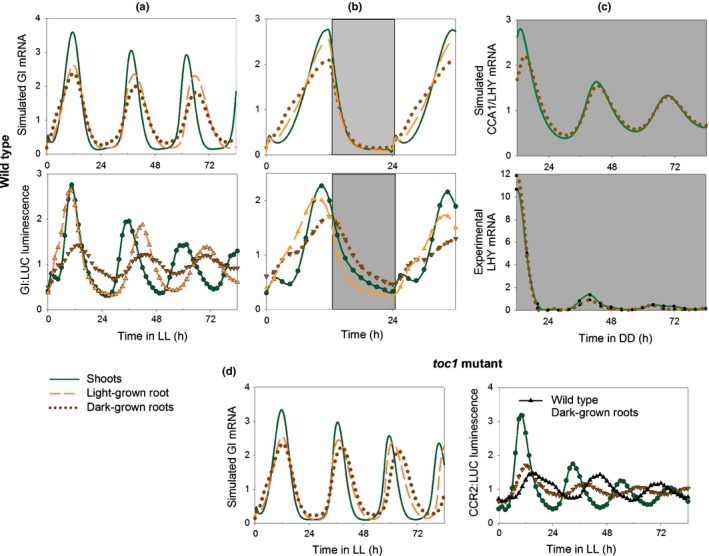
Different light inputs to the shoot and root clocks can simulate the differences and similarities observed between the two organs. The default values of parameters in the P2012 model were modified as listed in Supporting Information Table S6. Simulated mRNA (GIGANTEA (GI) and CIRCADIAN CLOCK ASSOCIATED 1 (CCA1)/LATE ELONGATED HYPOCOTYL (LHY)) data were compared with imaging (GI:LUC) or quantitative real‐time PCR (LHY mRNA) data in (a) light : light (LL), (b) light : dark (LD) and (c) dark : dark (DD) for wild‐type *Arabidopsis thaliana* plants and in LL for *timing of cab expression 1* (*toc1*) mutant plants (d). Filled triangles in (d) show experimental data for wild‐type dark‐grown roots to allow comparison with the *toc1* mutant. Both theoretical and experimental data were normalized to the mean values over the time‐courses. White bars, light; grey bars, dark.

Our new parameter sets gave a long FRP in both light‐ and dark‐grown roots compared with shoots, with the same period in shoots and roots under LD (24 h) and under DD (*c*. 27 h) (Fig. 8a–c). They also simulated lower amplitudes in dark‐grown roots compared with shoots in LL and in LD with intermediate values for light‐grown roots (Fig. 8a,b). In addition, they accurately described the different patterns of expression of *GI* between shoots and roots in LD: roots show a shoulder at dawn and a peak at dusk, while shoots show discrete peaks of *GI* at both dawn and dusk (Fig. 8b).

Next we simulated the behaviour of shoots and roots of the *toc1* mutant. The root model correctly described the longer FRP in roots than shoots and also the similar decreases of period in both organs of the *toc1* mutant compared with the wild‐type background; the simulated (for *GI*) and experimental (for *CCR2*) data are shown in Fig. 8(d). The root model also correctly described the shorter FRP (in LL) and earlier phase (in LD) in roots of the *cca/lhy* mutant compared with the wild‐type (Fig. S6). These data further validated the root model.

### The root model predicts organ‐specific response to skeleton photoperiods

As shoots and roots differ in light inputs, we predicted that the two organs would respond differently to skeleton photoperiods, a discriminating regime that comprises two short light treatments (e.g. 3 h) per daily cycle to entrain the clock. These conditions separate the contributions of light at dawn and at dusk which are merged in a complete photoperiod (Pokhilko *et al*., 2010). We used the P2011 model and our root parameters to simulate the responses of shoot and root clocks to the skeleton photoperiod of 3L : 6D : 3L : 12D.

**Figure 9:**
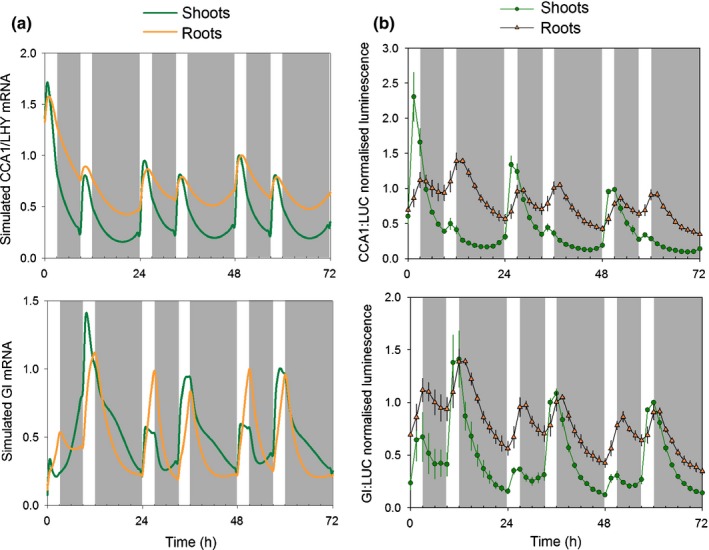
The shoot and root clocks respond differently to skeleton photoperiods. (a) Shoots were simulated with the default values of the P2011 model and dark‐grown roots with the values in Supporting Information Table S6. (b) Experimental data (four clusters of three plants imaged in two independent experiments for each genotype; mean ± SEM) were obtained for shoots and dark‐grown roots of *Arabidopsis thaliana* plants carrying the CIRCADIAN CLOCK ASSOCIATED 1:LUCIFERASE (CCA1:LUC+) or GIGANTEA (GI):LUC+ fusion, entrained for 3 wk under light : dark (LD, 12 h : 12 h) before transfer to skeleton photoperiods. Each time‐point was normalized to the mean luminescence level over the 72 h in the skeleton photoperiod. To compare peaks, both theoretical and experimental data were expressed relative to the highest values recorded over the last photoperiod (48–72 h). White bars, light; grey bars, dark.

Fig. 9(a) shows the simulations for *CCA1*/*LHY* and *GI* mRNA levels over 3 d of skeleton photoperiods following 3 wk of entrainment with regular 12L : 12D cycles. After a first transient day (time 0–24 h in Fig. 9a), the oscillations stabilized and clear differences between shoots and roots were evident. For *CCA1*/*LHY*, the amplitude of oscillation was lower in roots than shoots, consistent with the less acute activation of *CCA1*/*LHY* by light in the root model. *GI* peak levels in roots were similar at dawn and dusk, in shoots the *GI* dawn peak was much lower than its dusk peak, and at dawn the *GI* peak in shoots was slightly earlier than its peak in roots. This difference between shoots and roots can be explained by different levels of repressors at dawn in the two models. In the shoot model, the repression of *GI* expression by the CCA1/LHY protein at dawn limits the effect of an acute activation of *GI* by light in the early day. In the root model, the peak level of the CCA1/LHY protein is lower than in shoots (Fig. S12), allowing *GI* mRNA to continue rising in roots after 3 h of light at dawn.

Then we tested experimentally the predictions of the root model by measuring the bioluminescence of the CCA1:LUC and GI:LUC reporter genes (Fig. 9b). The model correctly predicted several features: the lower amplitude of *CCA1* in roots than shoots, the later dawn and dusk peaks of *CCA1* and *GI* in roots than in shoots and the similar levels of the dawn and dusk peaks of *GI* in roots compared with the different levels in shoots. The model did not correctly predict the relative heights of the dawn and dusk peaks in *CCA1* in shoots, nor the low amplitude of *GI* in roots. Nevertheless, it provided a qualitatively good prediction of the observed behaviour.

Our combination of theoretical and experimental work thus shows that in the plant circadian system light inputs are organ specific. This explains the differences and similarities between the shoot and root clocks under various experimental conditions (LL, LD, DD, and skeleton photoperiods) and in various genotypes (wild‐type and clock mutants). The work also demonstrates that light can directly entrain the root clock and may be more important than shoot‐derived signals in entrainment.

## Discussion

Temporal compartmentalization of biological processes is a key advantage of circadian clocks found in most organisms. In Arabidopsis, although the clocks of shoots and roots are in phase under LD cycles, they are desynchronized under LL (James *et al*., 2008). This was thought to be the consequence of different core clock structures in the two organs, with synchronization by a photosynthesis‐related signal in LD. Here, we propose a single mechanism that could explain both differences and similarities between the shoot and root clocks: organ‐specific light inputs within similar circadian systems.

Our earlier study of the root clock (James *et al*., 2008) was based on RT‐qPCR and microarray data. Here, we developed an imaging system to monitor simultaneously clock gene expression in shoots and roots. Imaging is nondestructive, which reduces the biological variability of the results, and allows improved estimation of period via increased time resolution and longer time‐courses. Thain *et al*. (2002) also imaged luciferase activity in roots, but under conditions of high light intensity and in the presence of sucrose. These conditions certainly allow higher signals in roots but are less relevant physiologically; for example, external supply of sucrose was shown to increase the amplitude of clock genes in shoots under DD and affected their FRP (Dalchau *et al*., 2011). To improve the signal/noise ratio from roots, we imaged mature plants with an extensive root system and limited light scattering between the shoot and root compartments (Fig. S1). By introducing an automated cover for the root compartment, we developed the ability to image either dark‐grown or light‐grown roots. The results obtained using our imaging protocol were different from those obtained by RT‐qPCR in some respects, for example the period estimates in LL and DD. In LL this was probably attributable to the lower light intensities used for imaging and is consistent with Aschoff's rule. Nevertheless, the FRPs were always longer in roots compared with shoots with each method. Overall, the imaging data greatly enhance our understanding of the root clock.

It has been generally assumed that circadian clocks share similar components in every plant cell (Harmer, 2009). However, the data of James *et al*. (2008) indicated that the clock in mature wild‐type roots only involved a subset of these components. Our results reconcile these two views by showing that the clocks of shoots and roots have similar compositions but different dynamic properties (FRP, amplitude and phase), and consequently rhythms in some clock components of dark‐grown roots can be obscured. Even though some root markers were not scored as rhythmic in all data sets, overall the data show that *TOC1* and *GI* are evening clock components in roots as well as in shoots (Figs 1, 2, 3). However, the amplitudes of their oscillations are lower in roots compared with shoots, as shown both by imaging (Fig. 1) and at the transcript level (Fig. 2). Imaging showed that periods are not only longer but also more variable in roots than shoots under LL (Fig. 1). Together, the higher variability in FRP under LL and the lower amplitudes in dark‐grown roots compared with shoots made rhythms harder to detect in roots compared with shoots, both here and in a previous study (James *et al*., 2008).

In this study, mutations of clock genes had similar effects on the dynamics of the shoot and root clocks (Figs 3, S6a). The FRP of CCR2:LUC luminescence in dark‐grown roots is shorter in the *toc1‐4* mutant than in the wild‐type but is longer in both genotypes than its FRP in the corresponding shoots. Thain *et al*. (2002) also observed period shortening in light‐grown roots of a *toc1* mutant. These results are in contrast to those of James *et al*. (2008), where the *toc1‐10* mutation did not seem to shorten the FRP of *LHY* transcript abundance in roots compared with the wild‐type. One likely cause of this apparent discrepancy is the difference in the methods used, with imaging of promoter activity being more precise and sensitive than transcript quantification. In addition, for the *toc1* mutant the root models describe a bigger difference in period between shoots and dark‐grown roots than between shoots and light‐grown roots (Fig. 8d). This may partly explain why James *et al*. (2008) did not detect period shortening in dark‐grown roots of *toc1* mutants. However, it is also possible that the genes assessed (*CCR2* and *LHY*) and mutant backgrounds (*toc1‐4* and *toc1‐10*) used contribute to the anomaly; *toc1‐4* is a null mutant in Col with a stop codon close to the N‐terminus (Para *et al*., 2007), while *toc1‐10* in Ws has a large deletion after S255 (Locke *et al*., 2006). Similarly, *toc1‐2* affects the period of both *CAB2* promoter activity and cytosolic Ca^2+^ rhythms, whereas *toc1‐1* only affects *CAB2* promoter activity (Xu *et al*., 2007).

Our data reveal three properties of the root clock system that provide the basis for our modelling. First, it is very similar to the shoot clock in DD. Though assessment of rhythmicity in DD, either by imaging or by RT‐qPCR, is complicated by rapid damping (Figs S8, S9), as expected for experiments carried out in the absence of sucrose (Dalchau *et al*., 2011), plants expressing GI:LUC+ provided interpretable data. Shoots and roots showed similar periods, either in the absence or in the presence of sucrose. Second, the root clock can be entrained by direct perception of low light in the absence of shoots. Third, its period depends on light quality, being much longer in blue than red light (Fig. 6). Previous studies suggested that seedling and root circadian clocks can be entrained by rhythmic endogenous sugar signals (James *et al*., 2008; Haydon *et al*., 2013), but the difference in period between shoots and roots is unaffected by sucrose (Fig. 6). Recent work shows that the shoot apex sends an unidentified signal to roots that maintains circadian synchrony within roots (Takahashi *et al*., 2015). While lack of this signal may account for the progressive loss of rhythmicity in decapitated roots, our data show that it can be overcome by direct exposure of roots to LD cycles even at intensities as low as 0.15 μmol m^−2^ s^−1^ (Fig. S7). It is also clear that roots are entrained by light in preference to shoot‐derived signals (Fig. 5).

In physiological conditions, roots can receive low levels of illumination, enriched in the red and far red, via light piping along plant tissue (Mandoli & Briggs, 1984; Sun *et al*., 2003, 2005) or the limited penetration of light into soil (Tester & Morris, 1987). Illumination at 0.15 μmol m^−2^ s^−1^, which can entrain detached roots (Fig. S7), is similar to the intensity that might be expected after piping of full sunlight through 3–4 cm of stem or root tissue, depending on wavelength (Sun *et al*., 2005). It is interesting to note that the behaviour of dark‐grown roots reported here and by James *et al*. (2008) – low‐amplitude rhythms of clock genes and a reduced number of rhythmic output genes – is reminiscent of the properties of shoots exposed to far‐red light (Wenden *et al*., 2011). We therefore suggest that the direct effect of light on roots described here may be physiologically significant and contributes at least partly to the synchronization of shoots and roots in LD cycles noted by James *et al*. (2008). However, sucrose also clearly affects the robustness of root rhythms and it seems likely that both light and sugar signals can affect the root clock. In addition, Matsuzaki *et al*. (2015) found that, in the field, temperature is a strong regulator of leaf gene transcription, and temperature may also entrain roots. Overall, the mechanisms and relative strengths of entraining signals in physiological conditions remain to be identified.

These considerations led us to conclude that at constant temperature the root and shoot clocks differ mainly in light inputs. Multiple light signalling pathways are required for correct biological timing in Arabidopsis (Dalchau *et al*., 2010). Although not all light signalling pathways are described in the P2011 model, many light‐related processes are captured in this model. Modifying some of them based on a few constraints (period, amplitude and trough levels of clock gene transcripts in roots) allowed us to simulate at least qualitatively most of our data and led to predictions that were verified experimentally. This confirmed that organ specificity in the plant circadian system could be attributable to different light inputs to the shoot and root clocks. The fact that light quality affects the periods of the root and shoot clocks in different ways (Fig. 6) is a direct illustration of such differences. Silva‐Navas *et al*. (2015) have also noted that the responses of root length to direct illumination depend on light quality and that several photoreceptors are involved in these responses. However, our work does not exclude other potential differences between the two clocks apart from light inputs.

## Author contributions

S.B, S.S., A.J.M. and H.G.N. planned research. S.B., S.S., J.L. and H.G.N. performed experiments and analysed data. S.B., S.S., A.J.M. and H.G.N. wrote the manuscript.

## Supporting information

Please note: Wiley Blackwell are not responsible for the content or functionality of any supporting information supplied by the authors. Any queries (other than missing material) should be directed to the *New Phytologist* Central Office.

**Fig. S1** Overview of the imaging protocol.
**Fig. S2** The expression of CCR2 in roots is affected by direct exposure to light.
**Fig. S3** Relative amplitudes in LD are lower in dark‐grown roots than shoots.
**Fig. S4** The expression of GI in roots is affected by direct exposure to light.
**Fig. S5** TOC1 is expressed rhythmically in Col‐0 roots.
**Fig. S6** The *cca1/lhy* double mutation affects the shoot and root clocks similarly.
**Fig. S7** The root clock can be entrained by very low light intensity.
**Fig. S8** Rhythms of GI:LUC+ expression in DD without sucrose.
**Fig. S9** Transcript levels of morning and evening genes in DD.
**Fig. S10** Rhythms of GI:LUC+ expression in DD with 1% sucrose.
**Fig. S11** Reducing the value of *L* in the P2011 model increases the FRP in LL and reduces the amplitude in LD.
**Fig. S12** Correlation between protein levels and mRNA troughs in shoots and roots.
**Table S1** Primers used for PCR and qPCR
**Table S2** Periods and RAE values of clock gene expression in shoots and roots
**Table S3** Periods and amplitudes of gene expression in shoots and roots by qPCR
**Table S4** Periods of GI:LUC luminescence in shoots and roots: effects of light quality and sucrose
**Table S5** Periods of gene expression in DD for shoots and roots by qPCR
**Table S6** Parameter values for shoot and root clock models
**Methods S1** Modelling method
