# Supplementary material for: Organ specificity in the plant circadian system is explained by different light inputs to the shoot and root clocks
Source: New Phytol. 2016 May 31;212(1):136–49. doi: 10.1111/nph.14024 (PMC5006879; doi:10.1111/nph.14024)
Supplement: Supplementary file 1 — Fig. S1 Overview of the imaging protocol. Fig. S2 The expression of CCR2 in roots is affected by direct exposure to light. Fig. S3 Relative amplitudes in LD are lower in dark‐grown roots than shoots. Fig. S4 The expression of GI in roots is affected by direct exposure to light. Fig. S5 TOC1 is expressed rhythmically in Col‐0 roots. Fig. S6 The cca1/lhy double mutation affects the shoot and root clocks similarly. Fig. S7 The root clock can be entrained by very low light intensity. Fig. S8 Rhythms of GI:LUC+ expression in DD without sucrose. Fig. S9 Transcript levels of morning and evening genes in DD. Fig. S10 Rhythms of GI:LUC+ expression in DD with 1% sucrose. Fig. S11 Reducing the value of L in the P2011 model increases the FRP in LL and reduces the amplitude in LD. Fig. S12 Correlation between protein levels and mRNA troughs in shoots and roots. Table S1 Primers used for PCR and qPCR Table S2 Periods and RAE values of clock gene expression in shoots and roots Table S3 Periods and amplitudes of gene expression in shoots and roots by qPCR Table S4 Periods of GI:LUC luminescence in shoots and roots: effects of light quality and sucrose Table S5 Periods of gene expression in DD for shoots and roots by qPCR Table S6 Parameter values for shoot and root clock models Methods S1 Modelling method [file NPH-212-136-s001.pdf]

Supporting Information

Methods S1: Modelling method

In order to model the root clock we used the third sbml version of the P2011 model (Pokhilko *et al*, 2012), available on the PlaSMo database (http://www.plasmo.ed.ac.uk); this model fits RNA data from rosette leaves of mature plants (Flis *et al*., 2015).

1. Model structure

The P2011 model, which is shown in Fig. 6 of the main text, was based on seedling data but also accurately described our shoot data. The model comprises a set of ordinary differential equations representing the interrelation of mRNA and protein levels for the genes LHY/CCA1, TOC1, PRR9, PRR7, NI, ELF3, ELF4, LUX, and GI, and the protein levels of ZTL, COP1 and protein complexes such as the evening complex (EC); CCA1 and LHY are partially redundant and treated as one entity. In this model external light is represented by the light function L whose value can be 0 (dark) or 1 (full light) depending on the light conditions simulated; brief twilights are simulated by L values between 0 and 1. Dark is represented by the parameter $D = 1 - L$. The value of L is not a linear description of light intensity but could represent the strength of light input.

Our data suggests that the core clock structure is similar in shoots and roots. In addition roots can perceive light, either by direct illumination or by light piping from exposed tissues. We therefore used the P2011 model including the light function to simulate the behaviour of the root clock.

2. Equations including light inputs

Light enters the P2011 model through the parameter L in the following equations:

$$\frac{dc_L^m}{dt} = q_1 L c_P + n_1 \frac{g_1^{\ a}}{g_1^{\ a} + (c_{P9} + c_{P7} + c_{NI} + c_T)^a} - (m_1 L + m_2 D) \cdot c_L^m \quad (1)$$

$$\frac{dc_L}{dt} = (p_2 + p_1 L) \cdot c_L^m - m_3 c_L - p_3 \frac{c_L^{\ c}}{c_L^{\ c} + g_3^{\ c}} \quad (2)$$

$$\frac{dc_P}{dt} = p_7 D \cdot (1 - c_P) - m_{11} c_P L \quad (3)$$

$$\frac{dc_{P9}^m}{dt} = L \cdot q_3 \cdot c_P + \frac{g_8}{g_8 + c_{EC}} (n_4 + n_7 \cdot \frac{c_L^{\ e}}{g_9^{\ e} + c_L^{\ e}}) - m_{12} c_{P9}^m \quad (4)$$

$$\frac{dc_{COP1c}}{dt} = n_5 - p_6 c_{COP1c} - m_{27} c_{COP1c} (1 + p_{15} L) \quad (5)$$

$$\frac{dc_{COP1n}}{dt} = p_6 c_{COP1c} - n_6 L \cdot c_P \cdot c_{COP1n} - n_{14} c_{COP1n} - m_{27} c_{COP1n} (1 + p_{15} L) \quad (6)$$

$$\frac{dc_{COP1d}}{dt} = n_{14} c_{COP1n} + n_6 L \cdot c_P \cdot c_{COP1n} - m_{31} (1 + m_{33} D) \cdot c_{COP1d} \quad (7)$$

$$\frac{dc_{EC}}{dt} = p_{26} c_{LUX} c_{E34} - m_{36} c_{EC} \cdot c_{COP1n} - m_{37} c_{EC} \cdot c_{COP1d} - m_{32} c_{EC} (1 + p_{24} \cdot L \cdot \frac{c_{Gn\_tot}^{\ \ d}}{g_7^{\ d} + c_{Gn\_tot}^{\ \ d}}) \quad (8)$$

$$\frac{dc_{ZTL}}{dt} = p_{14} - p_{12}Lc_{ZTL}c_G + p_{13}c_{ZG}D - m_{20}c_{ZTL} \tag{9}$$

$$\frac{dc_{ZG}}{dt} = p_{12}Lc_{ZTL}c_G - p_{13}c_{ZG}D - m_{21}c_{ZG} \tag{10}$$

$$\frac{dc_G^m}{dt} = Lq_2c_P + n_{12}\frac{g_{14}}{g_{14}+c_{EC}}\cdot\frac{g_{15}^e}{g_{15}^e+c_L^e} - m_{18}c_G^m \tag{11}$$

$$\frac{dc_{Gc}}{dt} = p_{11}c_G^m - p_{12}Lc_{ZTL}c_{Gc} + p_{13}c_{ZG}D - m_{19}c_{Gc} - p_{17}c_{E3c}c_{Gc} - p_{28}c_{Gc} + p_{29}c_{Gn} \tag{12}$$

In these equations:
c_i^m and c_i represent concentrations of mRNA and protein, respectively. The subscript "*i*" denotes LHY/CCA1 (L), PRR9 (P9), PRR7 (P7), the Night Inhibitor (NI), TOC1 (T), ELF3 (E3), ELF4 (E4), LUX, the Evening Complex (EC), GI (G), ZTL and its complex with GI (ZG), and the protein P;
a, *c*, *d* and *e* exponents are Hill coefficients;
n_j and m_j are rate constants of transcription and degradation, respectively;
g_j are Michaelis-Menten constants;
q_j are the rate constants of acute (P-dependent) light activation of transcription;
p_j are constants of translation, protein modification, protein complex formation and translocation between nucleus and cytoplasm.

In these equations twelve parameters are multiplied by *L* and are termed the "light-related parameters". They can be grouped in two categories, respectively acute and permanent effects of light on the clock:
- the "P-related parameters" are also multiplied by c_P, that is m_{11}, n_6, q_1, q_2 and q_3. They affect the clock after a dark to light transition but not in constant light (LL) when c_P rapidly tends to 0;
- the other light-related parameters are multiplied by L but not by c_P. They all represent rate constants related to light: m_1 (degradation of CCA1/LHY mRNA), m_{27} (degradation of COP1n), m_{32} (degradation of the EC), p_1 (translation of CCA1/LHY mRNA), p_{12} (ZTL-GI complex formation), p_{15} (translocation of COP1 between nucleus and cytoplasm), and p_{24} (modification of EC). They are termed the "*L*-related parameters" and affect the clock whenever light is present.
The values of these parameters are presented in Supplementary Table S3. When $L = 0$, none of these parameters have an influence on clock dynamics except m_{27} and m_{32}, so these two parameters were not changed in the optimisation process.

3. Simulations

We used COPASI (version 4.8.35) to change the default (shoot) value of parameters related to light inputs (see below) and run simulations; the level of GI mRNA served as the main marker to compare experimental and theoretical data. To model the light function we used two step functions (Adams *et al.*, 2012): step function1 to simulate light:dark cycles (LD), LL and constant dark (DD) by setting two of its parameters (the "amplitude step1" and the "offset step1") to 0 or 1 as shown in Supplementary Table S3, and step function2 to simulate skeleton photoperiods. Mutations of clock genes were simulated by decreasing the rates of gene transcription to zero. For each new set of parameters the simulations were first run for

20 d under LD (which corresponds to 3 wk old plants) before release into other condition (e.g. LL or DD).

4. Parameter change strategy

Our experimental data suggests that light inputs to the clock are organ-specific and may explain the different dynamics observed in shoots and roots. To test this hypothesis and to fit our root clock data we changed the value of L and light-related parameters with the following constrains (ranked in order of decreasing importance):
- longer periods in light- and dark-grown roots compared to shoots under LL
- lower amplitudes in dark-grown roots compared to shoots under LD
- higher trough levels for evening genes in dark-grown roots compared to shoots

We noticed that reducing the value of L gives a longer free-running period (FRP) in LL (Fig. S11a). (Changing the value of L to x requires changing the parameter D to D = x-L for simulations in LD, so that D reaches the value 0 during light cycles.) This reproduces the observation that period lengthens in LL when the light intensity is reduced, consistent with Aschoff's rules. In addition the amplitude of transcript levels are reduced when $L = 0.5$ (Fig S11a), which reproduces our observations in dark-grown roots compared to shoots (Fig. 1). We therefore decided to set L to 0.5 and 1 to simulate dark- and light-grown roots respectively. Since this single change could not reproduce all our root data we then modified other light-related parameters as described below.

The concentration of the protein P (c_P) rapidly tends to 0 when light is on so the "P-related parameters" have virtually no influence on the dynamics of the clock under LL. Therefore these parameters did not have to be changed to fit the longer FRP of roots compared to shoots. However our experimental data showed that the expression of GI and PRR9, but not CCA1, was acutely induced in roots exposed to light (Fig. 1b). Therefore the value of q_1, which represents the acute activation of CCA1/LHY mRNA transcription by light, was halved. This change also allowed a more accurate representation of the phase of CCA1/LHY expression under LD cycles (when $L = 0.5$) (Fig. S11b).

The "L-related parameters" (m_1, p_1, p_{12}, p_{15} and p_{24}) were changed individually and in combination. Each of these parameters was first scanned individually with COPASI by increasing or decreasing their default value up to 10-fold, and then at higher resolution over a 2-fold range. None of these single changes gave a satisfactory fit to the root data (e.g. if the period was long enough, the oscillation dampened out). Therefore combinations of parameters had to be changed simultaneously.

Starting from the default values of the P2011 model (except that the values of L and q_1 were halved as above) we decided to focus on the parameters p_1, p_{15} and p_{24} for the following reasons:
- The changes to L and q_1 could simulate dark-grown root data satisfactorily (Fig. S11b) but the FRP in LL was still too short (< 26 h), and some amplitudes were too high and/or troughs were too low.
- Troughs are generally higher in roots, especially for evening genes (Fig. S6B). Given the structure and equations of the P2011 model we reasoned that the trough values could be increased if the levels of the LHY protein or the EC were lower (Fig. S12a). This could be achieved by decreasing p_1 and p_{15} and by increasing p_{24}.
- The system was extremely sensitive to changes in m_1 but insensitive to changes in p_{12}, so their values were not changed initially.

Many combinations of p_1, p_{15} and p_{24} were tested as follows:
- The FRP should be ~27 h, i.e. 2 h more than the shoot FRP in the model. Experimentally, these values may vary depending on the technique used and the initial conditions, but the difference between shoot and root periods is typically 2 h or more.
- Satisfactory sets of parameters were then tested in the same condition (LL) but with $L = 1$ to simulate light-grown roots; the FRP should still be higher than in shoots
- these sets of parameters were also tested in LD to check whether the amplitude was lower in dark-grown roots compared to light-grown roots and shoots.

Our optimal combination of parameters was the following: q_1, p_1 and p_{15} were halved and p_{24} was increased by 50%. This gave a longer FRP in dark- ($L = 0.5$) and light-grown roots ($L = 1$) compared to shoots and lower amplitudes in roots compared to shoots. To further increase the FRP in light-grown roots and reach a similar value as dark-grown roots, the value of m_1 was decreased by 25% for light-grown roots (i.e. with $L = 1$). These new parameter sets for roots also fitted experimental data that had not been used in the optimisation, such as the effects of mutation on periods (Fig. 7) and the organ-specific response to skeleton photoperiod (Fig. 8).

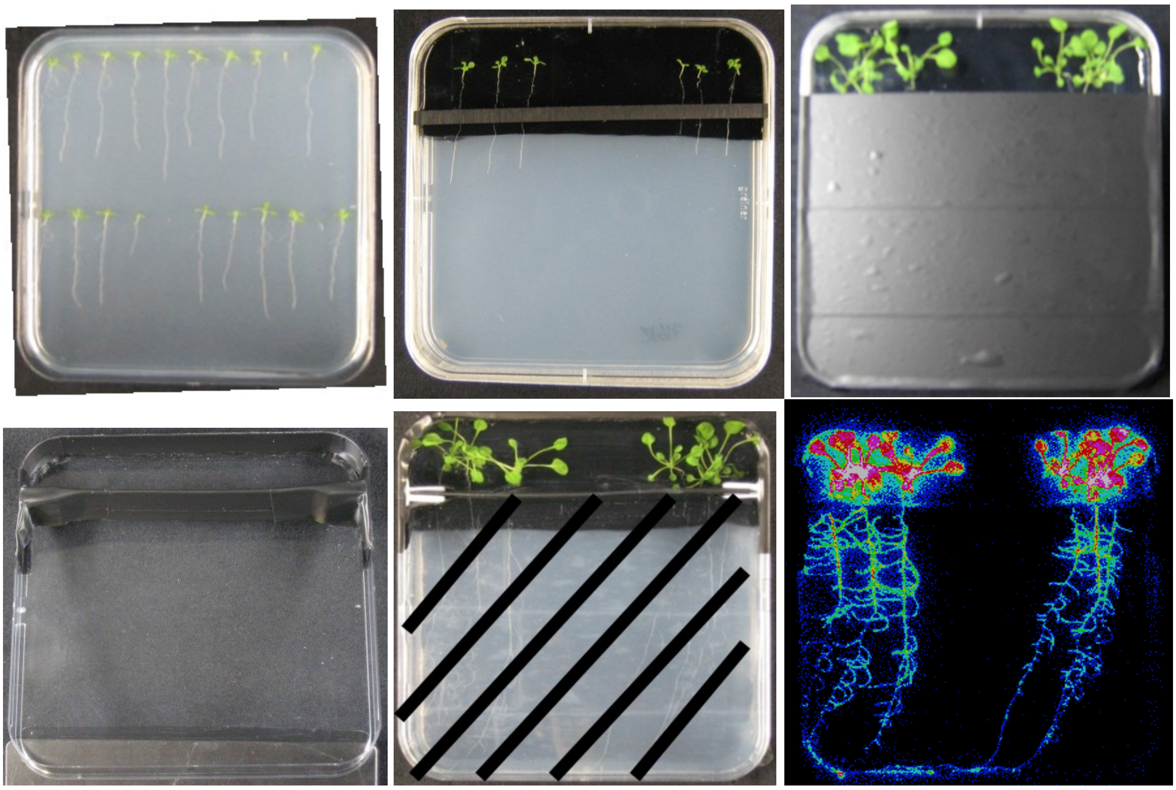

Figure S1: Brief overview of the optimised imaging protocol, from sowing to imaging

Seedlings were grown on 1/2 MS medium for 10-12 d (top left) before being transferred on fresh 1/2 MS medium with charcoal (top middle). Roots were then either light- or dark-grown. In the latter case, a black acrylic bar was placed horizontally under the shoots (top middle) to minimise light leakage to the roots; to keep the roots in the dark, black tape was placed around the plate (top right). After another 10-12 d of entrainment in LD (12/12) at 20º C, plants were ready for imaging (top right). The original lid (with black tape, or without if roots were light-grown) was removed. The plants were sprayed with luciferin (~6 µmol per plant) and the new lid (bottom left) was used to seal the plate. This new lid contained a black barrier and tape that distinguished two compartments (one for shoots and one for roots) to reduce light scattering between the two organs. An example of a bright field image and the corresponding luminescence are shown in the bottom middle and bottom left panels respectively. For dark-grown roots, a shutter (represented by hatched bars, bottom middle) was placed over the root compartment when the lights were on.

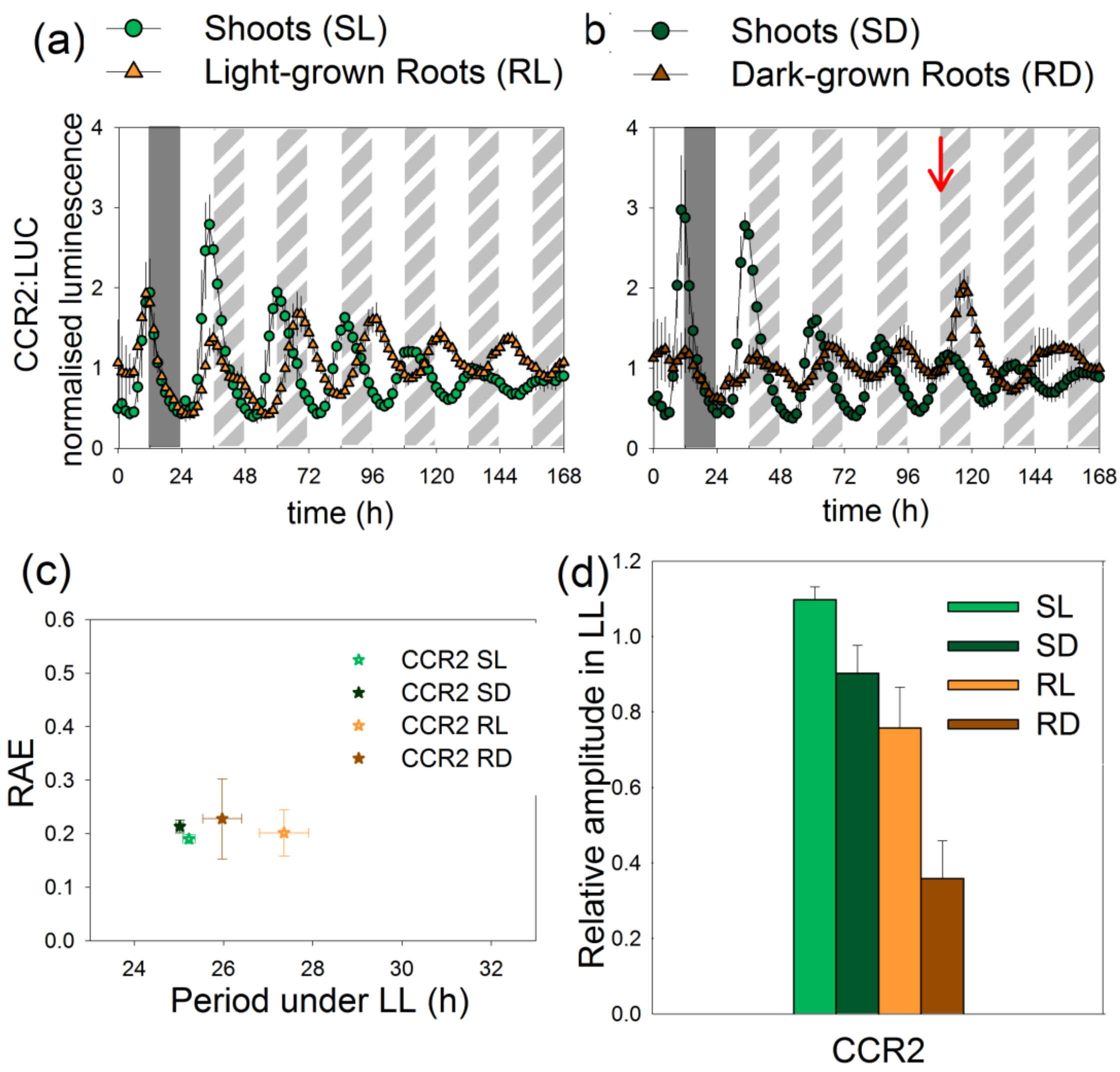

Figure S2: The expression of CCR2 in roots is affected by direct exposure to light

Plants carrying the CCR2:LUC+ fusion were entrained for 3-4 wk in LD before release into LL. The promoter activities in plants with light-grown (a) and dark-grown (b) roots were monitored over the last d in LD (time 0 = dawn) and in LL. From 108 h (red arrow in (b)), dark-grown roots were exposed to the same light conditions as shoots and light-grown roots. For each experiment, 1-3 clusters of 2-6 plants (organs) were imaged separately and luminescence data were normalised to the mean values between 0 and 108 h. Error bars are +SEM for 3 independent experiments. The backgrounds show d or subjective d (white bars), night (dark grey bars) and subjective night (hatched bars). Circadian periods (c) and relative amplitudes (d) were estimated between 48 and 108 h for individual time-courses (S = Shoots, R = Roots; L= Light-grown roots and D= Dark-grown roots). Relative amplitudes were normalised to the mean relative amplitude of the shoots (SL and SD). Error bars are +SEM.

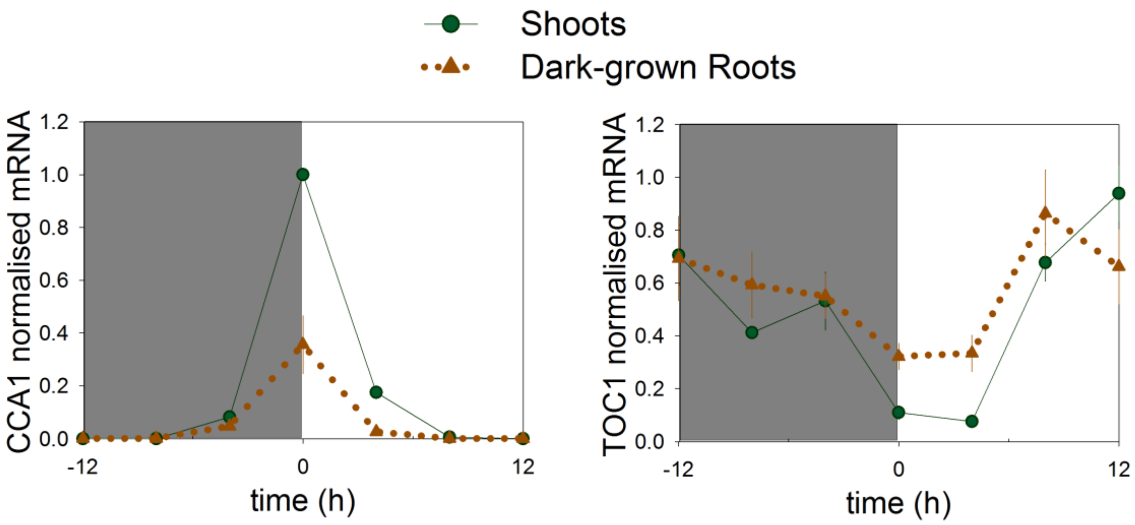

Figure S3: Relative amplitudes in LD are lower in dark-grown roots than shoots

Plants were grown in hydroponic culture with dark-grown roots. Plants were entrained for 4 wk in LD before harvesting from dusk (ZT -12). mRNA levels of *CCA1* (left) and *TOC1* (right) were measured in shoots and roots. The experiment was repeated 3 times independently. For each experiment mRNA levels were normalised to *ISU1*, expressed to the highest value in shoots over the corresponding time course and then averaged. Error bars are ± SD. The backgrounds show d (white bar) and night (dark grey bar).

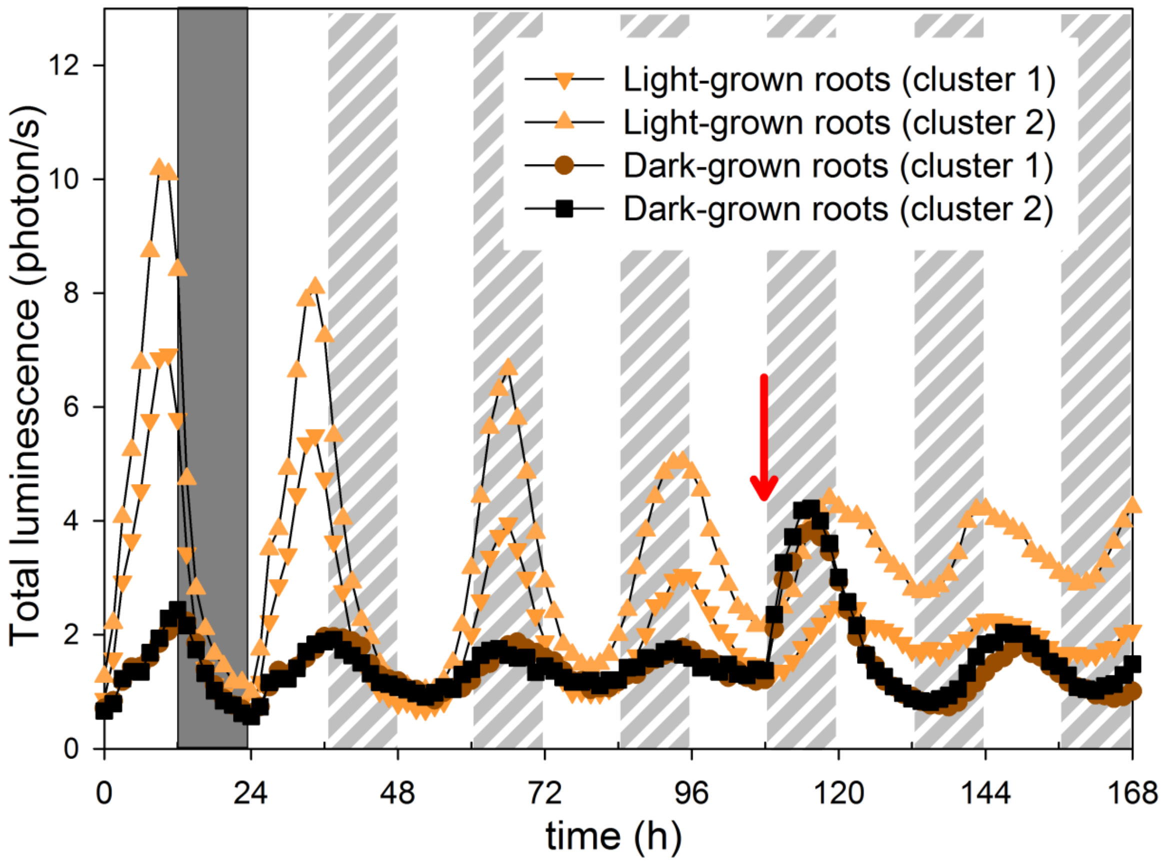

Figure S4: The expression of GI in roots is affected by direct exposure to light

Plants carrying the GI:LUC+ fusion were entrained for 3-4 wk in LD before release into LL. The promoter activities in plants with light-grown (a) dark-grown (b) roots were monitored over the last d in LD (ZT0 = dawn) and in LL. From ZT108 (shown by the red arrow), dark-grown roots were exposed to the same light conditions as shoots and light-grown roots. The experiment was repeated 3 times independently. Fig. 1 shows normalised data from these experiments. Here the raw data of 4 clusters of 2-6 plants are shown. The backgrounds show d or subjective d (white bars), night (dark grey bar) and subjective night (hatched bars).

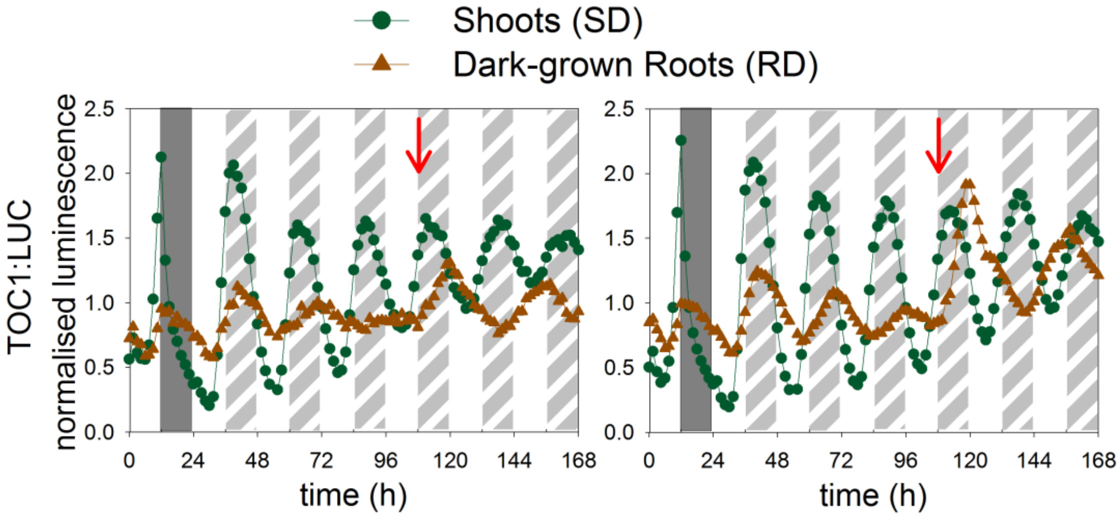

Figure S5: TOC1 is expressed rhythmically in Col-0 roots

Col-0 plants carrying the TOC1:LUC+ fusion were entrained for 3-4 wk in LD before release into LL. The promoter activities in plants with dark-grown roots were monitored over the last d in LD (ZT0 = dawn) and in LL. From time 108 h (red arrows), roots were exposed to the same light conditions as shoots. Conditions are as in Fig. 1 but with a different ecotype. One cluster of 3-6 plants was imaged in two independent experiments. Data from individual time-courses were normalised to their mean values between time 0 and 108 h. Bars in the backgrounds represent d or subjective d (white bars), night (dark grey bars) and subjective night (hatched bars). Mean circadian periods were estimated between 48 and 108 h as 24.14 h and 27.60 h for shoots and roots respectively.

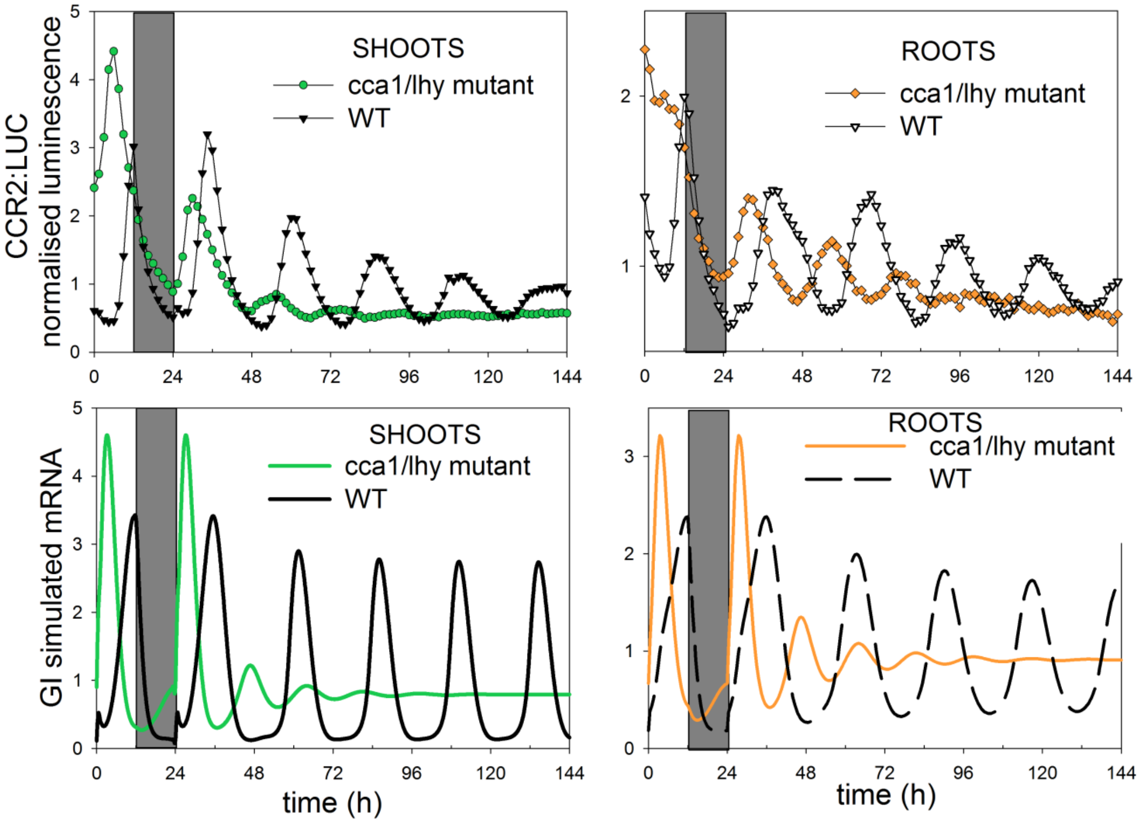

Figure S6: The *cca1/lhy* double mutation affects the shoot and root clocks similarly

Experimental data (top) and simulations (bottom) for shoots and dark-grown roots in wild type and mutant plants. *cca1/lhy* double mutant plants carrying the *CCR2:LUC*+ reporter were entrained for 5 wk in LD cycles before imaging. They were then imaged for 1 d in LD and 5 d in LL. Simulations of GI mRNA were used for comparison with the experimental data of the evening gene CCR2. Shoots and dark-grown roots were simulated in the wild type with the same parameters as in Figs. 7-8, and the *cca1/lhy* mutant was simulated by setting the values of n_1 and q_1 to 0. Each trace was normalised to the mean over the whole time-course. The dark grey bars in the backgrounds represent the last night before release into LL.

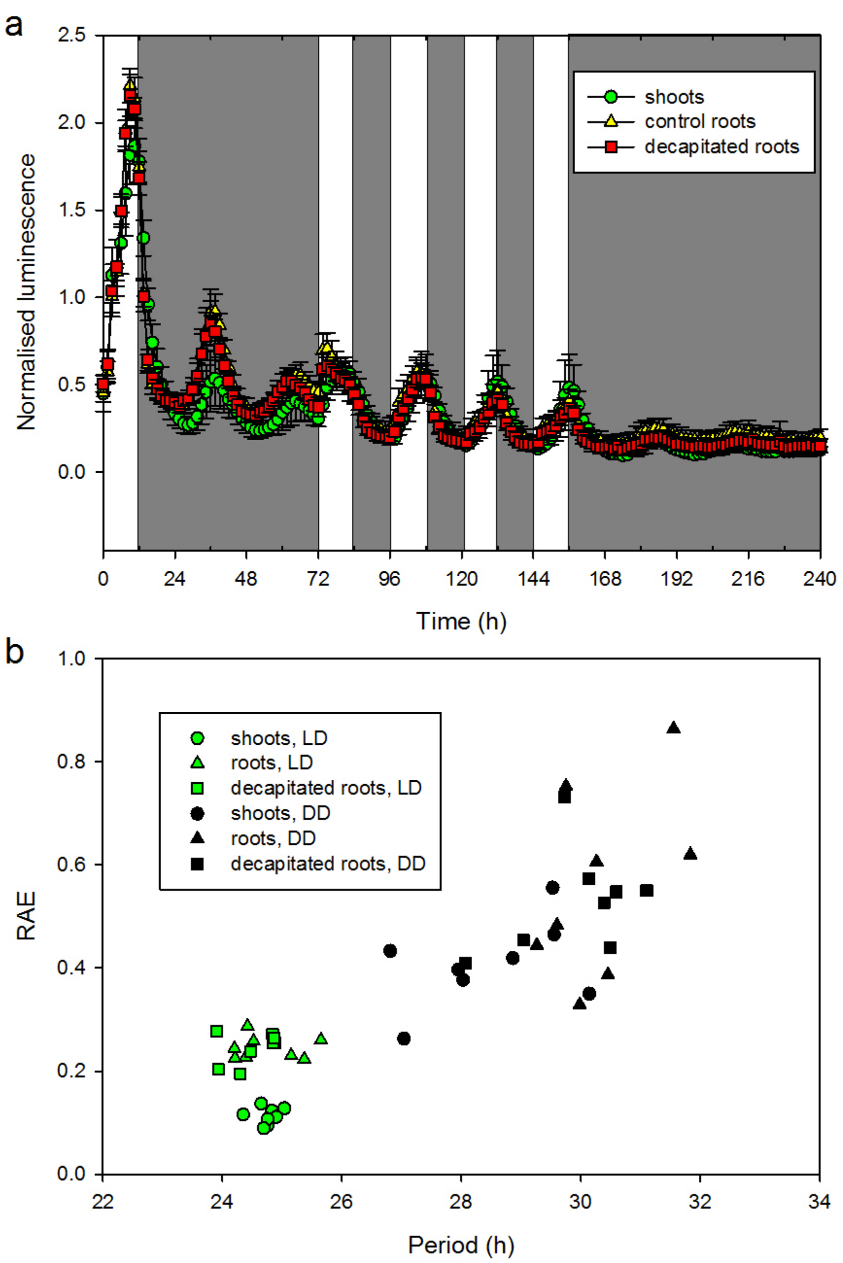

Figure S7: The root clock can be entrained by very low light intensity

Plants with dark-grown roots carrying the GI:LUC+ reporter were sprayed with luciferin, maintained for 2 further LD cycles (12 h light : 12 h dark) at 15 µmol.m^{-2}.s^{-1}, for 48 h in DD, over 4 LD cycles at 0.15 µmol.m^{-2}.s^{-1} (equal intensities of red and blue light) and finally in DD. Imaging commenced at the start of the second LD cycle. Some plants were decapitated 24 h before this. Periods were estimated for 96-168 h (LD) and 180-240 h (DD). Panel (a) - time courses, means $\pm$ SD for n=8 clusters of plants from 2 biological replicates. Panel (b) – assessment of period against relative amplitude error (RAE) for the individual traces summarised in panel (a).

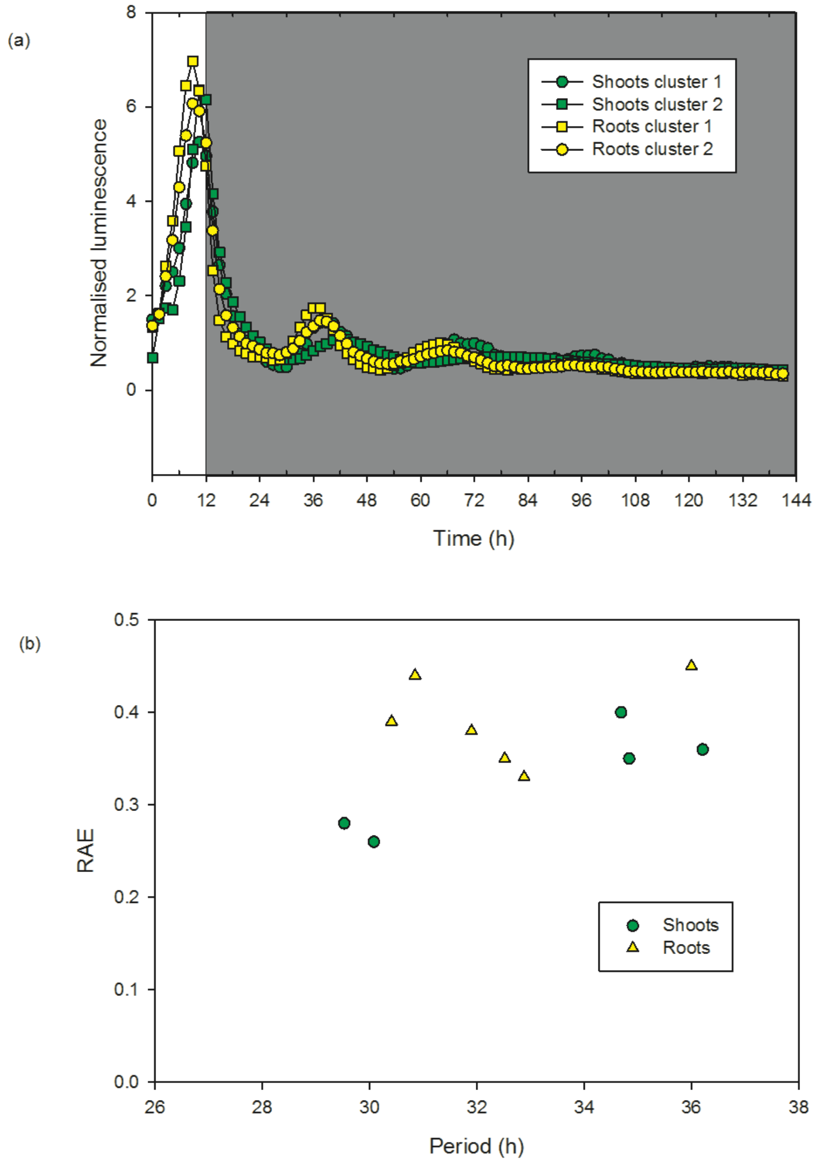

Figure S8: Rhythms of GI:LUC+ expression in DD without sucrose

Plants with light-grown roots carrying the GI:LUC+ reporter were entrained for 3-4 wk in LD (white light) before imaging over 24 h in LD followed by DD. Panel (a) shows representative traces for individual clusters of plants, panel (b) shows periods and RAE values estimated over 72 h in DD (48-120 h in (a)) for individual clusters of plants that were scored rhythmic in two separate experiments. Mean periods ± SD were: roots, 32.4 ± 2.0 h (n=6); shoots, 33.1 ± 3.0 h (n=5), P>0.1 by Student's t-test.

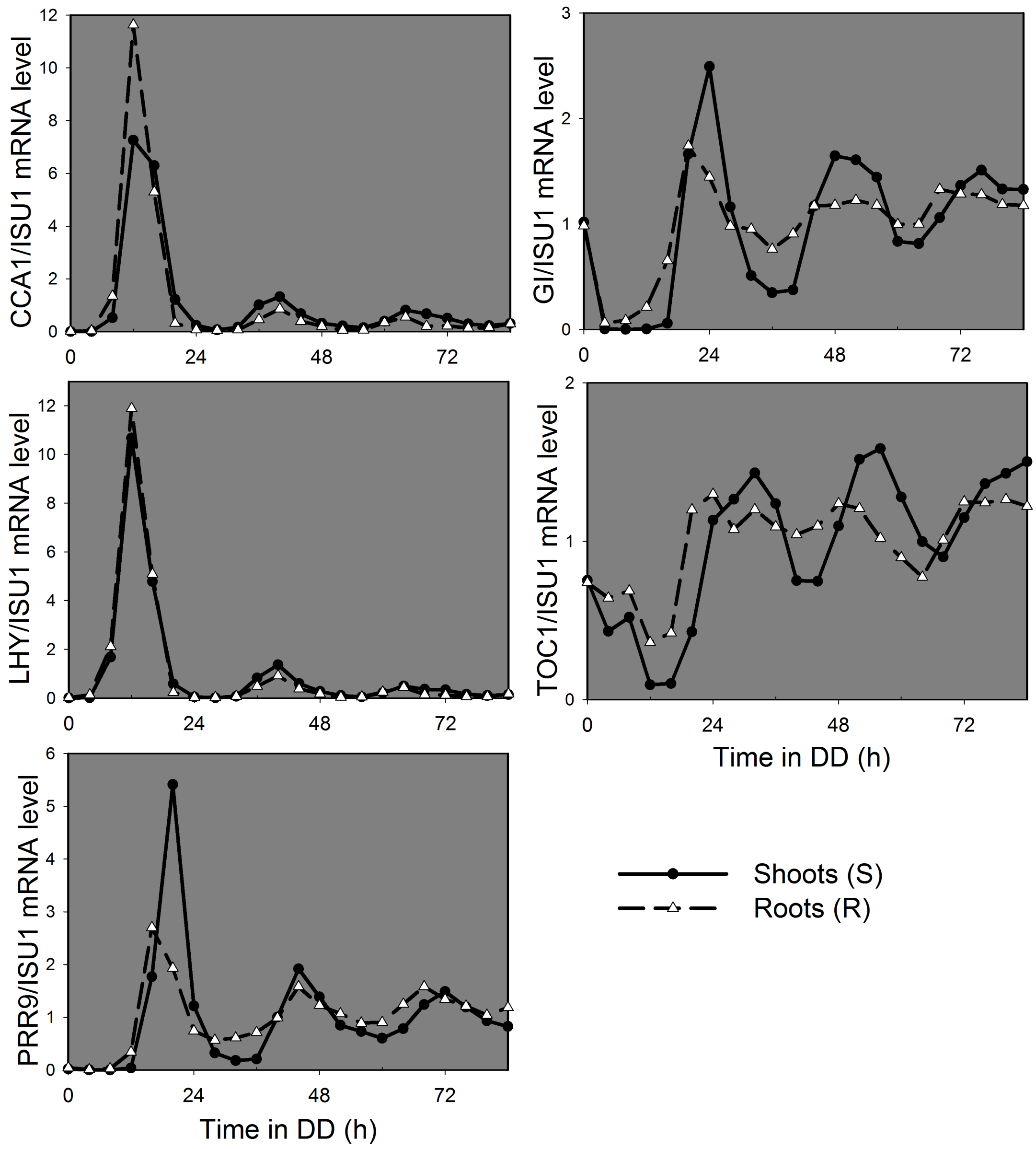

Figure S9: Transcript levels of morning and evening genes free run in DD with similar FRPs in shoots and roots

Plants were grown in hydroponic culture with dark-grown roots. Plants were entrained for 4 wk in LD before release in DD. mRNA levels of *CCA1*, *LHY* and *PRR9* (left) and *GI* and *TOC1* (right) in shoots and roots; mRNA levels are normalised to *ISU1*. Time 0 is the last dusk before DD. Periods are shown in Table S5.

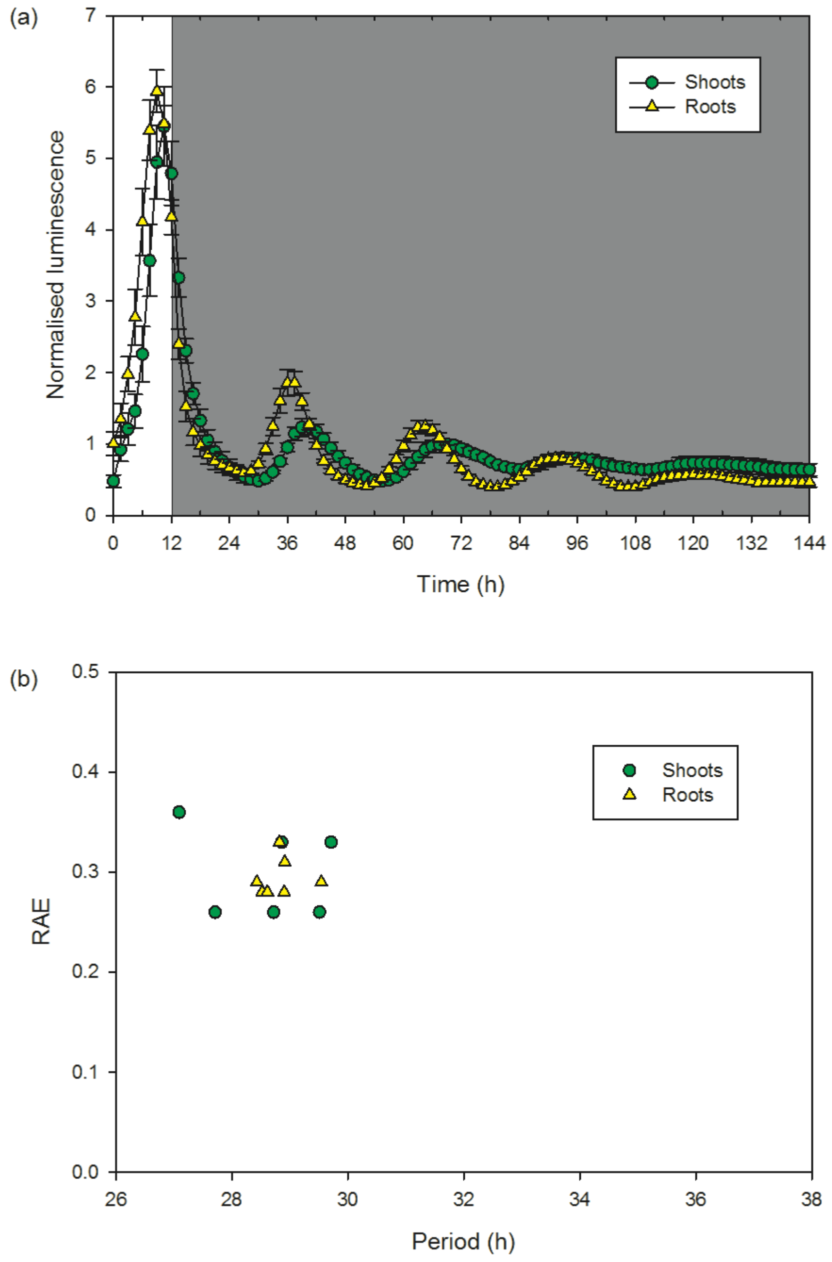

Figure S10: Rhythms of GI:LUC+ expression in DD with 1% sucrose

Plants with light-grown roots carrying the GI:LUC+ reporter were entrained on plates containing 1% sucrose for 3-4 wk in LD (white light) before imaging. (a): Promoter activities were monitored for 6 clusters of plants (shoots) and 7 clusters (roots) in two separate experiments, data are means $\pm$ SD. (b): periods and RAEs were measured with BRASS using the data from 48 to 120 h in (a). The mean periods (shoots, 28.8 $\pm$ 1.0 h, roots 28.6 $\pm$ 0.4 h) were not significantly different by Student's t-test (P>0.4).

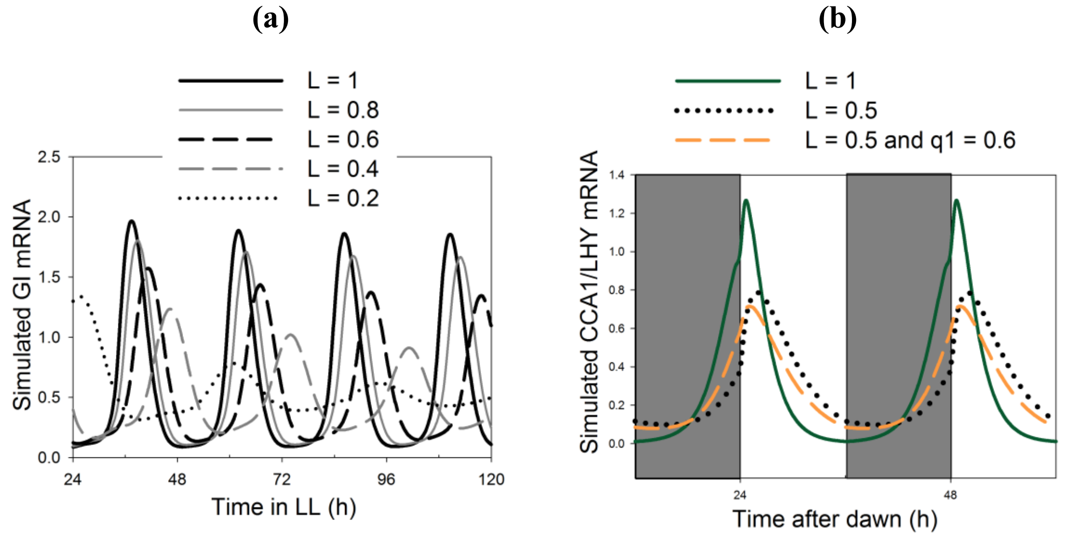

Figure S11: Reducing the value of *L* in the P2011 model increases the FRP in LL and reduces the amplitude in LD

Simulations of *GI* (a) and *LHY/CCA1* (b) mRNA in LL and LD respectively. The parameter *L* was scanned with COPASI between 0.2 and 1 in LL (a). In LD simulations are shown for $L = 1$ and $L = 0.5$ with the default value of q_1, and $L = 0.5$ with q_1 halved (set to 0.6).

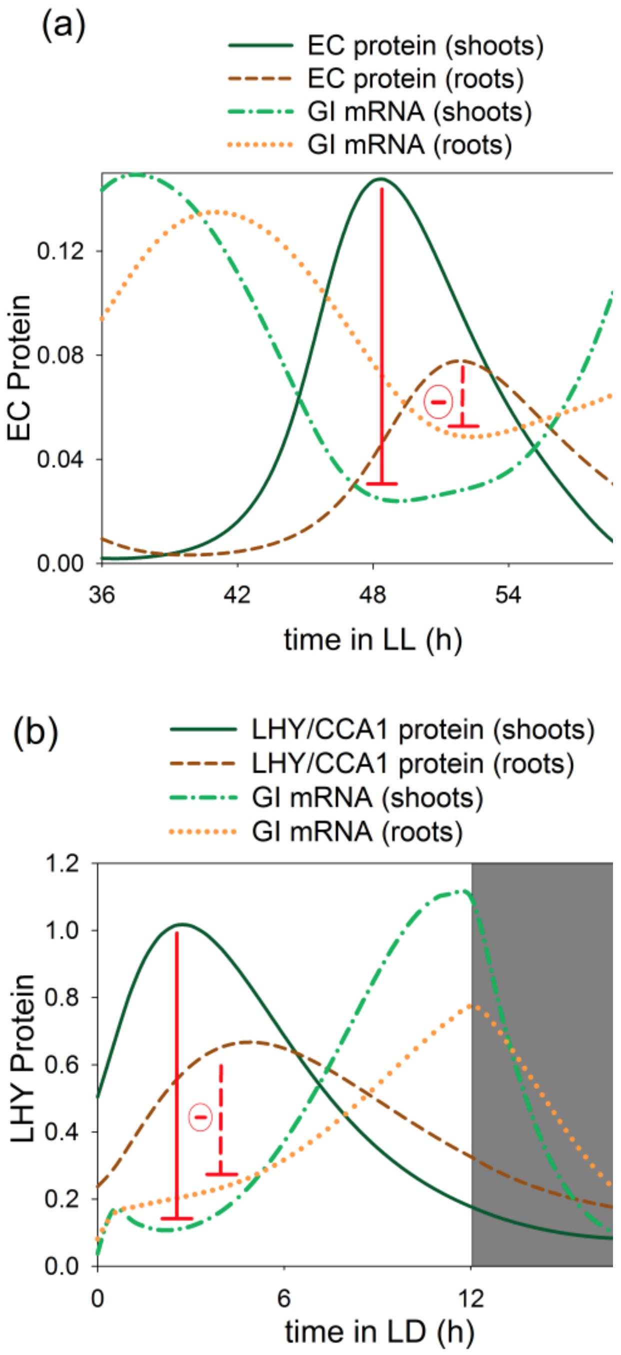

Figure S12: Correlation between protein levels and mRNA troughs in shoots and roots

Protein and mRNA levels were simulated using the P2011 model, with the default parameters for shoots and the modified parameters described in Fig. 7 and table S4 for dark-grown roots
(a) Simulation of EC protein and GI mRNA in LL
(b) Simulation of LHY/CCA1 protein and GI mRNA in LD
Proteins are represented by dark green lines (shoots) or brown dashes (roots). GI mRNA is represented by light green dash-dot (shoots) or orange dots (roots). Vertical red lines and dashes represent inhibition of transcription by proteins in shoots and roots respectively.

Table S1: Primers used for PCR and qPCR

Target	Forward	Reverse
Actin (PCR)	CTTACAATTTCCCGCTCTGC	GTTGGGATGAACCAGAAGGA
CCA1	ATCTGGTTATTAAGACTCGGAAGCC	GCCTCTTTCTCTACCTTGGAGAAAA
LHY	GAATTATTAGCTAAGGCAAGAAAGCC	GCCTCTTTCTCCAACTTTGTGAAGA
TOC1	GTGTTCTTATCAAGTGACTGCAGTG	CAAGTCCTAGCATGCGTCTTCTTC
PRR7	GTCTTTAAGTGCTTATCGAAAGGAGC	CACTACCACTAGAACTTTGGCATCT
PRR9	TGTTGAAGTGTATGCTGAGAGGTGC	ATCATCACGCAAAGTCAGTCTTCTC
CHS	CAGACAGGACATCGTGGTGGT	ACATGAGTGATCTTTGACTTGG
GI	CGGGCAACTGATGGAATGCTTG	TTGTTGCTGGTAGACGACACTTC
ISU1	GCCATCGCTTCTTCATCTGTTGC	GTGGGAGAGAAAGATGCTTTGCG

Table S2: Periods and RAE values of clock gene expression in shoots and roots

Values were estimated between times 48 and 108 h for the individual time-courses used for Figs. 1a and 1b and are shown graphically in Fig. 1c; S = Shoots, R = Roots; L= Light-grown roots, D= Dark-grown roots.

promoter	organ	PERIOD		RAE	
		mean	s.e.m.	mean	s.e.m.
GI	SL	24.31	0.08	0.17	0.01
	SD	24.46	0.33	0.19	0.01
	RL	28.85	0.25	0.22	0.01
	RD	26.41	0.15	0.15	0.04
TOC1	SL	24.33	0.19	0.13	0.01
	SD	23.93	0.30	0.17	0.02
	RL	28.53	0.19	0.31	0.02
	RD	30.02	1.48	0.49	0.04
PRR9	SL	24.30	0.29	0.22	0.04
	SD	23.90	0.11	0.15	0.01
	RL	30.80	1.36	0.41	0.04
	RD	25.41	0.80	0.39	0.05
CCA1	SL	24.28	0.16	0.14	0.01
	SD	24.79	0.15	0.13	0.03
	RL	27.51	1.63	0.43	0.03
	RD	25.60	1.12	0.28	0.03

Table S3: Periods and amplitudes of gene expression in shoots and roots by qPCR

Periods and relative amplitudes were estimated between times 24 and 84 h for the individual time-courses whose means are shown in Fig. 2 (S = Shoots, R = Roots; L= Light-grown roots, D= Dark-grown roots). Relative amplitudes were normalised to the mean relative amplitude of the shoots (SL and SD data combined) for each reporter. Values are means ± SD for n = 2 biological replicates.

	promoter	SL	SD	RL	RD
Period	CCA1	23.8 + 0.5	23.5 + 1.1	25.8 + 0.3	26.3 + 0.1
	LHY	23.6 + 0.3	23.5 + 0.7	25.9 + 0.2	26.9 + 1.0
	PRR9	23.0 + 0.6	22.3 + 0.7	25.4 + 0.1	25.5 + 0.5
	GI	23.0 + 0.2	23.2 + 0.1	25.2 + 0.3	26.3 + 0.1
	TOC1	22.9 + 0.4	23.1 + 0.6	27.1 + 0.3	29.1 + 0.3
Normalised relative amplitude	CCA1	1.02 + 0.05	0.98 + 0.05	0.91 + 0.01	0.82 + 0.10
	LHY	1.02 + 0.05	0.98 + 0.05	0.82 + 0.01	0.74 + 0.09
	PRR9	1.06 + 0.09	0.94 + 0.10	0.74 + 0.05	0.72 + 0.06
	GI	1.12 + 0.12	0.88 + 0.11	0.70 + 0.11	0.42 + 0.07
	TOC1	1.05 + 0.05	0.95 + 0.05	0.45 + 0.03	0.30 + 0.02

Table S4: Periods of GI:LUC luminescence in shoots and roots: effects of light quality and sucrose

Light	Red	Blue
Roots control	28.2 ± 0.2 h	31.8 ± 1.1 h*
Roots plus sucrose	27.6 ± 0.4 h	29.5 ± 0.9 h**
Shoots control	26.0 ± 0.5 h	24.6 ± 0.6 h*
Shoots plus sucrose	25.6 ± 0.5 h	24.8 ± 0.2 h

Periods were assessed using BRASS from the individual traces summarised in Fig. 6. Values are means ± SD for n=4 clusters of plants in 2 biological replicates.
Differences in period between red and blue light were assessed by Student's t-test, *, P<0.05, ** P< 0.01

Table S5: Periods of gene expression in DD for shoots and roots by qPCR

Values were estimated using BRASS over the times 24-84 h in the time-courses shown in Fig. S9.

Promoter	Shoot period (h)	Root period (h)
CCA1	26.0	24.5
LHY	26.2	24.7
PRR9	26.1	24.9
GI	27.1	25.3
TOC1	26.7	27.4

Table S6: Parameter values for shoot and root clock models

The default parameters of the P2011 model were used except as shown below. *, in the day *L* = 0.5 or 1 except for brief twilight periods at dawn and dusk.

Organ	Condition	Amp Step1	Offset Step1	L	D	q_1	p_1	p_{15}	p_{24}	m_1
**Shoots**	LD	1	0	0-1*	1-L	1.2	0.13	3	10	0.54
	LL	0	1	1	1-L	1.2	0.13	3	10	0.54
	DD	0	0	0	1-L	1.2	0.13	3	10	0.54
**Light grown roots**	LD	1	0	0-1*	1-L	0.6	0.065	1.5	15	0.405
	LL	0	1	1	1-L	0.6	0.065	1.5	15	0.405
**Dark grown roots**	LD	0.5	0	0-0.5*	0.5-L	0.6	0.065	1.5	15	0.54
	LL	0	0.5	0.5	0.5-L	0.6	0.065	1.5	15	0.54
	DD	0	0	0	0.5-L	0.6	0.065	1.5	15	0.54

Reference List

Adams RR, Tsorman N, Stratford K, Akman OE, Gilmore S, Juty N, Le Novere N, Millar AJ, Millar AJ. (2012). The Input Signal Step Function (ISSF), a Standard Method to Encode Input Signals in SBML Models with Software Support, Applied to Circadian Clock Models. *Journal of Biological Rhythms* **27**: 328-332.

Flis A, Fernandez AP, Zielinski T, Mengin V, Sul;pice S, Stratford K, Hume A, Pokhilko A, Southern MM, Seaton DD *et al.* (2015). Defining the robust behaviour of the plant clock gene circuit with absolute RNA timeseries and open infrastructure. *Open Biology* **5**: 150042

Pokhilko A, Fernandez AP, Edwards KD, Southern MM, Halliday KJ, Millar AJ. (2012). The clock gene circuit in Arabidopsis includes a repressilator with additional feedback loops. *Molecular Systems Biology* **8**:574.
